# Natural variants modify *Klebsiella pneumoniae* carbapenemase (KPC) acyl–enzyme conformational dynamics to extend antibiotic resistance

**DOI:** 10.1074/jbc.RA120.016461

**Published:** 2020-12-03

**Authors:** Catherine L. Tooke, Philip Hinchliffe, Robert A. Bonomo, Christopher J. Schofield, Adrian J. Mulholland, James Spencer

**Affiliations:** 1 School of Cellular and Molecular Medicine, Biomedical Sciences Building, University of Bristol, Bristol, United Kingdom; 2 Centre for Computational Chemistry, School of Chemistry, University of Bristol, Bristol, United Kingdom; 3 Louis Stokes Cleveland Department of Veterans Affairs Medical Center, Cleveland, Ohio, USA; 4 Departments of Medicine, Pharmacology, Molecular Biology and Microbiology, Biochemistry, and Proteomics and Bioinformatics, Case Western Reserve University School of Medicine, Cleveland, Ohio, USA; 5 CWRU-Cleveland VAMC Center for Antimicrobial Resistance and Epidemiology (Case VA CARES) Cleveland, Ohio, USA; 6 Chemistry Research Laboratory, Department of Chemistry, University of Oxford, Oxford, United Kingdom

**Keywords:** serine β-lactamase, acyl–enzyme, enzyme catalysis, structure–function, crystal structure, molecular dynamics, β-lactam, antibiotic resistance, ceftazidime, DW, water molecule in the putative deacylating position, ESBLs, extended-spectrum class A β-lactamases, ESOCs, expanded-spectrum oxyimino-cephalosporins, GNB, Gram-negative bacteria, KPC, *Klebsiella pneumoniae* carbapenemase, MD, molecular dynamics, RMSD, root-mean-square deviation, RMSF, root mean square fluctuation, SBLs, serine β-lactamases

## Abstract

Class A serine β-lactamases (SBLs) are key antibiotic resistance determinants in Gram-negative bacteria. SBLs neutralize β-lactams *via* a hydrolytically labile covalent acyl–enzyme intermediate. *Klebsiella pneumoniae* carbapenemase (KPC) is a widespread SBL that hydrolyzes carbapenems, the most potent β-lactams; known KPC variants differ in turnover of expanded-spectrum oxyimino-cephalosporins (ESOCs), for example, cefotaxime and ceftazidime. Here, we compare ESOC hydrolysis by the parent enzyme KPC-2 and its clinically observed double variant (P104R/V240G) KPC-4. Kinetic analyses show that KPC-2 hydrolyzes cefotaxime more efficiently than the bulkier ceftazidime, with improved ESOC turnover by KPC-4 resulting from enhanced turnover (*k*_cat_), rather than altered *K*_M_ values. High-resolution crystal structures of ESOC acyl–enzyme complexes with deacylation-deficient (E166Q) KPC-2 and KPC-4 mutants show that ceftazidime acylation causes rearrangement of three loops; the Ω, 240, and 270 loops, which border the active site. However, these rearrangements are less pronounced in the KPC-4 than the KPC-2 ceftazidime acyl-enzyme and are not observed in the KPC-2:cefotaxime acyl-enzyme. Molecular dynamics simulations of KPC:ceftazidime acyl-enyzmes reveal that the deacylation general base E166, located on the Ω loop, adopts two distinct conformations in KPC-2, either pointing “in” or “out” of the active site; with only the "in" form compatible with deacylation. The "out" conformation was not sampled in the KPC-4 acyl-enzyme, indicating that efficient ESOC breakdown is dependent upon the ordering and conformation of the KPC Ω loop. The results explain how point mutations expand the activity spectrum of the clinically important KPC SBLs to include ESOCs through their effects on the conformational dynamics of the acyl–enzyme intermediate.

Increasing antimicrobial resistance threatens global public health (1), exemplified by resistance to β-lactams (*e.g.* carbapenems and cephalosporins), the most prescribed antibiotics worldwide (2). Cephalosporins, first introduced in the 1960s, remain among the most widely used antibiotics, with dozens of compounds differing in their substituents and antimicrobial properties (3). Resistance to cephalosporins and other β-lactams in Gram-negative bacteria (GNB) is predominantly mediated by production of β-lactamases (1), which catalyze hydrolysis of the β-lactam ring, rendering the antibiotic inactive. β-Lactamases are divided into four groups based on sequence and mechanism (4, 5, 6): classes A, C, and D are active-site serine enzymes (serine β-lactamases [SBLs]) that hydrolyze β-lactams *via* a covalent acyl–enzyme intermediate, whereas class B metallo-β-lactamases use zinc cofactors (6). Carbapenems are the most recently introduced β-lactam antibiotic class, with potent activity toward GNB, and inhibit many SBLs through formation of long-lived and hydrolytically inert acyl-enzymes (2, 7). However, their increasing use has resulted in the emergence of carbapenemases, β-lactamases that hydrolyze carbapenems, threatening their continued clinical efficacy (8, 9). Indeed, carbapenem-resistant Enterobacteriaceae and *Acinetobacter* spp. were identified by the Centers for Disease Control and Prevention as one of five urgent antibiotic resistance threats (10).

*Klebsiella pneumoniae* carbapenemase-2 (KPC-2), a class A SBL, was isolated from a carbapenem-resistant *K. pneumoniae* strain collected in 1996 (11). Sequence variants were discovered soon after (12, 13), and KPC family members have now been identified on multiple mobile genetic elements, enabling dissemination in numerous pathogenic GNB, such as *Escherichia coli*, *Pseudomonas aeruginosa* (14), *Klebsiella* spp., and *Acinetobacter* spp. (15). Unlike extended-spectrum class A β-lactamases (ESBLs) such as CTX-M-15 (16), KPC-2 is capable of hydrolyzing almost all β-lactam antibiotics, including carbapenems and most cephalosporins (17). The endemic status of KPC SBLs worldwide, together with their hydrolytic activity against a broad spectrum of β-lactams, makes this enzyme family a leading cause of carbapenem and other β-lactam failure in health care–associated infections. The KPC family now comprises up to 54 distinct enzymes (18) representing various insertions, deletions, and substitutions.

The parent enzyme, KPC-2, efficiently hydrolyzes penicillins, cephalosporins, and carbapenems but poorly hydrolyzes the expanded-spectrum oxyimino-cephalosporin (ESOC), ceftazidime, particularly compared with the structurally similar cefotaxime (Fig. 1) (13, 17). Cefotaxime and ceftazidime feature a C7 oxyimino substituent (Fig. 1), hindering binding and turnover by narrow-spectrum SBLs, such as SHV-1 and TEM-1 (19, 20), and demonstrate broad-spectrum activity against Gram-positive and Gram-negative aerobic and anaerobic bacteria (21, 22). Ceftazidime, for example, is an effective treatment for infections by organisms including *E. coli*, *K. pneumoniae*, and *P. aeruginosa* (22). Compared with cefotaxime, ceftazidime has a more complex, bulkier, C7 substituent with an additional carboxylate and two methyl groups and is consequently poorly accommodated by the active sites of most SBLs, including carbapenemases such as KPC-2. Consistent with this, for most SBLs that turn over ceftazidime, steady-state *K*_M_ values are in the high micromolar to millimolar range (17, 23, 24, 25). Ceftazidime is now combined with the SBL inhibitor avibactam (Avycaz (26) or Zavicefta (27)) to bolster clinical efficacy against resistant SBL producers, particularly Enterobacteriaceae and drug-resistant *P. aeruginosa* (26, 27). Avycaz has been approved to treat bacterial pneumonia and complicated urinary tract and intra-abdominal infections (26, 27).

**Figure 1:**
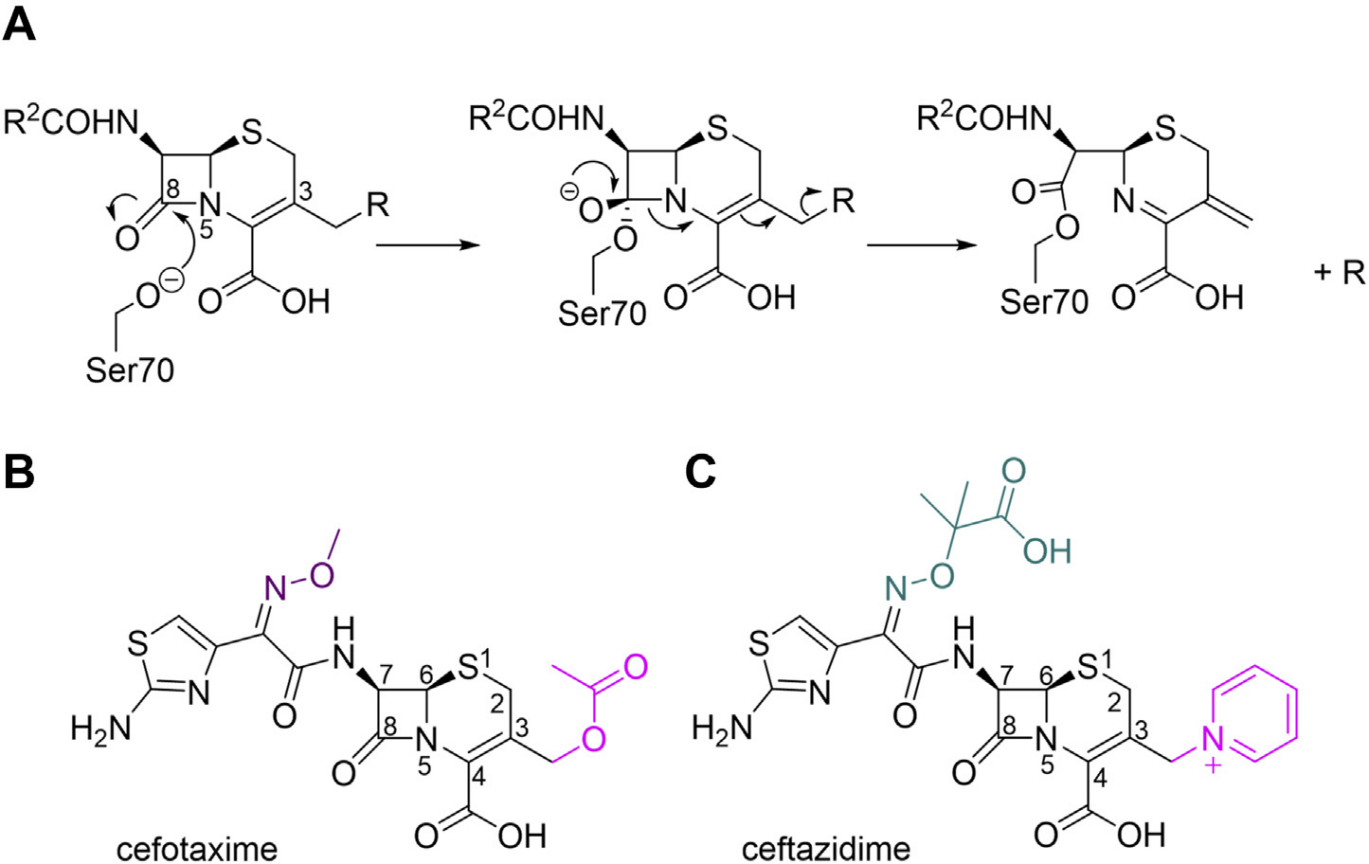
**Structure and reaction of β-lactam antibiotics.***A*, acylation of cephalosporins. *B* and *C*, structures of cefotaxime and ceftazidime. The C7 side chain oxyimino groups are colored *purple* (methoxyimino for cefotaxime) and *teal* (2-carboxypropan-2-yloxyimino for ceftazidime). C3' leaving groups (pyridinium for ceftazidime) or (acetoxy for cefotaxime) are colored *pink*.

KPC variants are the focus of increasing attention, particularly with respect to activity towards ceftazidime. The most comprehensive study to date investigated the activity and stability of the natural KPC variants 2 to 11 that possess single- or double-point mutations at four positions (M49I, P104R, P104L, V240G, V240A, and H274Y; Table S1 (13)). This identified differences in ceftazidime hydrolysis, with the variants KPC-4 (P104R and V240G substitutions) and KPC-10 (P104R and H274Y) exhibiting 50- and 75-fold increases in *k*_cat_/*K*_M_ compared with KPC-2, resulting in 32- and 42-fold increases in the ceftazidime minimal inhibitory concentration when the respective variants are expressed in recombinant *E. coli* (13). A reduction in thermodynamic stability of these variants further indicated that increased ceftazidime hydrolysis may be at a stability cost (13). Avycaz resistance, which appeared soon after its first clinical use in 2015, is linked to the emergence of KPC variants, some of which are thought to impact ceftazidime kinetics (28, 29, 30).

Despite recent structural characterization of KPC enzymes, including several complexes with inhibitors (31, 32) and two with antibiotic hydrolysis products (33), their interactions with substrates are not well understood. In particular, the absence of structures for any acyl–enzyme complexes limits explanation of their unusually broad spectrum of activity, and of how naturally occurring point variants tune activity toward different substrates (13). Here we describe investigations of ESOC turnover by KPC-2 and its doubly substituted variant KPC-4 that seek to understand the basis for the enhanced ceftazidime turnover, and consequent ceftazidime resistance of producer bacteria, by the latter. Crystal structures of the ceftazidime and cefotaxime acyl-enzymes of KPC-2, and the ceftazidime acyl-enzyme of KPC-4, identify that KPC-2 undergoes more extensive rearrangement on ceftazidime acylation than does KPC-4; extended molecular dynamics (MD) simulations of the acyl–enzyme complexes identify a conformation of the Ω loop, favored in KPC-2, that orients E166, the general base for deacylation, away from the acyl–enzyme carbonyl, restricting deacylation. These results highlight the mobility of loops within and around the KPC active-site pocket and the importance of this to hydrolysis of the ESOC substrate ceftazidime. Effecting alterations to the dynamic properties of the acyl–enzyme intermediate then provides a mechanism by which naturally occurring enzyme variants can modulate specificity.

## Results

### V240G and P104R substitutions in KPC-4 improve turnover of oxyimino-cephalosporins

To investigate activity differences between the naturally occurring KPC variants KPC-2 and KPC-4 (P104R:V240G), we first determined steady-state kinetic parameters for their hydrolysis of the oxyimino-cephalosporins ceftazidime and cefotaxime as well as the reporter substrate nitrocefin (34) (Table 1).

**Table 1:** Steady-state parameters for cephalosporin hydrolysis by KPC-2 and KPC-4

Substrate	Kinetic parameter	KPC-2	KPC-4
Ceftazidime	*k*_cat_ (s^−1^)	1.9 (0.12)	81 (7.6)
	*K*_M_ (μM)	530 (70)	640 (110)
	*k*_cat_/*K*_M_ (μM^−1^ s^−1^)	0.0035 (5.2 × 10^−4^)	0.13 (0.025)
Cefotaxime	*k*_cat_ (s^−1^)	76 (6.6)	262 (32)
	*K*_M_ (μM)	200 (29)	190 (49)
	*k*_cat_/*K*_M_ (μM^−1^ s^−1^)	0.38 (0.064)	1.37 (0.039)
Nitrocefin	*k*_cat_ (s^−1^)	610 (9)	170 (44)
	*K*_M_ (μM)	18 (5)	59 (20)
	*k*_cat_/*K*_M_ (μM^−1^ s^−1^)	34 (9.3)	3 (1.2)

KPC, *Klebsiella pneumoniae* carbapenemase.

Standard errors are provided in parentheses; n = 3; calculated in GraphPad Prism.

Consistent with previous reports of increased catalytic efficiency (*k*_cat_/*K*_M_) for ceftazidime hydrolysis by some KPC variants (13), we observe a 40-fold increase of *k*_cat_/*K*_M_ for KPC-4 compared with KPC-2. Ceftazidime *K*_M_ values are similarly high for both variants (530 μM for KPC-2 and 640 μM for KPC-4), the turnover rate increases 40-fold (*k*_cat_ 1.9 s^−1^ for KPC-2 and 81 s^−1^ for KPC-4). The improved efficiency of ceftazidime hydrolysis by KPC-4 can therefore be attributed to changes in turnover, rather than *K*_M_. The two KPC variants also differ in their turnover of other cephalosporins (cefotaxime and nitrocefin). For cefotaxime, a substrate resembling ceftazidime, but with a smaller C7 group (Fig. 1), *K*_M_ values are lower than those for ceftazidime for both KPC-2 and KPC-4 (200 and 190 μM, respectively) and *k*_cat_ values substantially faster (76 and 262 s^−1^), resulting in KPC-4 ~fourfold more efficiently hydrolyzing cefotaxime than KPC-2 (*k*_cat_/*K*_M_ 1.37 μM^−1^ s^−1^ compared with 0.38 μM^−1^ s^−1^). Conversely, for nitrocefin, a nonclinical reporter substrate (34) similar to earlier cephalosporins, *k*_cat_/*K*_M_ for KPC-2 is tenfold higher than for KPC-4 (34 μM^−1^ s^−1^ and 3 μM^−1^ s^−1^, respectively) reflecting differences in Michaelis constant (*K*_M_ = 18 and 59 μM, respectively) and turnover rate (*k*_cat_ = 610 and 170 s^−1^, respectively). Thus, compared with KPC-2, the P104R/V240G double substitutions in KPC-4 selectively improve activity against bulkier ESOCs through acceleration of turnover rate (*k*_cat_).

### Deacylation-deficient KPC-2 and KPC-4 mutants yield crystal structures of cephalosporin acyl-enzymes

To further study the basis for KPC activity against the ESOCs cefotaxime and ceftazidime, deacylation-deficient mutants of KPC-2 and KPC-4 were generated by conservative substitution of the general base E166 (35) with the isosteric residue Gln (KPC-2^E166Q^ and KPC-4^E166Q^). Exposure of both KPC-2^E166Q^ and KPC-4^E166Q^ crystals to cephalosporins allowed trapping of the respective acyl-enzymes. We obtained high-resolution crystal structures of the acyl-enzymes of KPC-2^E166Q^ with both ceftazidime and cefotaxime (resolution of 1.25 and 1.31 Å, respectively) and of KPC-4^E166Q^ with ceftazidime (resolution of 1.24 Å). Data collection and refinement statistics are presented in Table 2. Clear *F*_o_ − *F*_c_ difference density in the KPC active sites allowed confident modeling of the Ser70 acyl-enzymes, with ligand real-space correlation coefficients >0.95 (Fig. S1 and Table S2). Uninterrupted polypeptide chains starting at residue 23 (KPC-2^E166Q^, KPC-2^E166Q^:ceftazidime, and KPC-4^E166Q^:ceftazidime) or 25 (KPC-4^E166Q^ and KPC-2^E166Q^:cefotaxime) to residue 294 were built into the experimental electron density for all structures except KPC-2^E166Q^:ceftazidime, for which residues 166 to 172 and 270 to 274 were not well resolved.

**Table 2:** Crystallographic data collection and refinement statistics

Dataset	KPC-2^E166Q^	KPC-2^E166Q^:cefotaxime	KPC-2^E166Q^:ceftazidime	KPC-4^E166Q^	KPC-4^E166Q^:ceftazidime
PDB ID	6Z21	6Z23	6Z24	6Z22	6Z25
Data collection					
Beamline	DLS I24	ALBA XALOC-BL-13	DLS I04	DLS I04	DLS I04
Space group	*P*2_1_2_1_2	*P*2_1_2_1_2	*P*2_1_2_1_2	*P*2_1_2_1_2	*P*2_1_2_1_2
Molecules/ASU	1	1	1	1	1
Cell dimensions					
a, b, c (Å)	60.20, 78.44, 55.96	60.14, 78.91, 55.65	60.27, 77.21, 55.41	60.20, 78.94, 55.98	60.23, 77.26, 55.74
α, β, γ (°)	90, 90, 90	90, 90, 90	90, 90, 90	90, 90, 90	90, 90, 90
Resolution (Å)	45.56–1.30 (1.32–1.30)	45.48–1.31 (1.33–1.31)	77.21–1.25 (1.27–1.25)	45.66–1.40 (1.42–1.40)	55.74–1.24 (1.26–1.24)
*R*_pim_	0.034 (0.686)	0.028 (0.770)	0.028 (0.315)	0.052 (0.696)	0.038 (0.828)
CC1/2	0.999 (0.460)	0.999 (0.638)	0.999 (0.833)	0.999 (0.674)	0.999 (0.747)
I/σ (I)	11.8 (0.9)	12.4 (0.9)	14.5 (2.2)	8.8 (1.3)	9.4 (1.0)
Completeness (%)	100 (100)	100 (100)	99.2 (98.0)	100 (100)	98.6 (97.0)
Redundancy	12.6 (12.2)	12.6 (13.1)	12.9 (12.4)	13.4 (12.3)	13.8 (14.3)
Refinement					
Resolution (Å)	32.86–1.30	45.48–1.31	55.41–1.25	45.66–1.40	55.571–1.24
No. of reflections	65,793	64,287	71,368	53,103	73,046
Rwork/Rfree	0.143/0.173	0.1601/0.1792	0.1497/0.1798	0.1417/0.1763	0.1453/0.1704
No. of atoms					
Protein	2087	2133	2029	2063	2132
Solvent	279	251	285	337	294
Ligand	—	52	31	—	62
*B*-factors (Å^2^)					
Protein	22	24	16	20	18
Solvent	41	45	37	35	38
Ligand	—	32	17	—	23
RMSD					
Bond lengths (Å)	0.008	0.009	0.011	0.007	0.010
Bond angles (°)	0.982	1.063	1.389	0.954	1.141
Ramachandran (%)					
Outliers	0.00	0.00	0.00	0.00	0.00
Favoured	98.9	98.48	98.81	98.5	98.5

ASU, asymmetric unit; DLS, Diamond Light Source; ID, identity; KPC, *Klebsiella pneumoniae* carbapenemase; PDB, Protein Data Bank; RMSD, root-mean-square deviation.

Values in parentheses represent information from the highest resolution shells.

We also determined structures of uncomplexed KPC-2^E166Q^ (resolution of 1.30 Å) and KPC-4^E166Q^ (resolution of 1.40 Å), confirming that mutation at position 166 does not induce substantial changes in the overall fold of the native enzyme (KPC-2 Protein Data Bank [PDB] 5UL8 (33), root-mean-square deviation [RMSD] = 0.157 Å and KPC-4 6QWE (31), RMSD = 0.201 Å for KPC-2^E166Q^ and KPC-4^E166Q^, respectively; Fig. S2 and Table S3). There are few differences between the active sites of KPC-2 and KPC-2^E166Q^. The hydrogen bond networks, observed in most class A SBL structures, which involve S70, K73, S130, E166, N170 and an active-site water molecule in the putative deacylating position (labeled DW; Fig. S2 and Table S4), remain unperturbed. In KPC-4, however, a small change in the position of Q166 (compared with E166 in the unmodified enzyme) results in repositioning (by ∼2.4 Å) of the putative deacylating water. The positions of other active-site residues are identical to those observed in native KPC-4 (Fig. S2*B* and Table S4).

**Figure 2:**
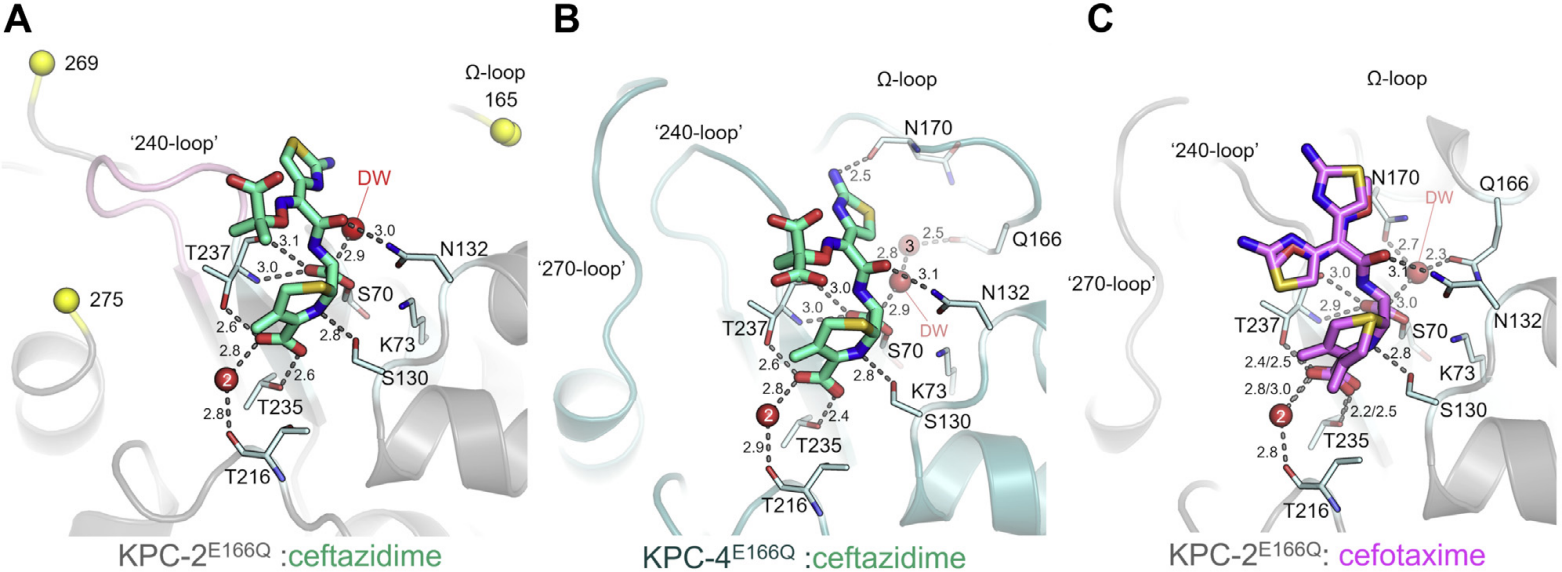
**Oxyimino-cephalosporin acyl–enzyme complexes with KPC-2**^**E166Q**^**and KPC-4**^**E166Q**^. Ceftazidime is represented as *green cyan* sticks and cefotaxime as *pink* sticks. The secondary structure is displayed as a cartoon, for KPC-2^E166Q^ in *gray* and KPC-4^E166Q^ in *teal*. Regions not modeled because of incomplete or poor electron density are flanked by *yellow* spheres representing the *C*_α_ atoms of the last residue modeled. *A*, *B*, and *C,* interactions of cefotaxime and ceftazidime within the KPC-2^E166Q^ and KPC-4^E166Q^ active sites. Key amino acids are highlighted as *pale blue* sticks, water molecules as *red* spheres, and distances as *dashes*. KPC, *Klebsiella pneumoniae* carbapenemase.

Ceftazidime is bound in one clearly resolved conformation in the KPC-2^E166Q^ acyl–enzyme complex and was refined at full occupancy (Fig. S1*A*). However, in KPC-4^E166Q^, the 2-carboxypropan-2-yloxyimino group of the ceftazidime C7 substituent (Fig. 1*B*; *teal*) was modeled in two conformations: major (A, 75% occupancy) and minor (B, 25% occupancy) in which the orientations of the carboxylate and methyl groups differ by 180° (Fig. S1*B* and Table S2). Similarly, cefotaxime was refined in complex with KPC-2^E166Q^ in two conformations (A, 49% and B, 51%; Fig. S1*C* and Table S2) that differ largely through a ∼180° rotation of the oxyimino group and aminothiazole ring of the C7 substituent. This is associated with movement of the cephem dihydrothiazine ring, which shifts to prevent a clash with the reoriented aminothiazole ring in conformation A (Figs. 2*C* and S1*C*). The electron density of all three KPC acyl-enzymes was not observed for the C3 substituents (Fig. S1), indicating that the 3' pyridinium (ceftazidime) groups or acetoxy (cefotaxime) groups have been efficiently eliminated after acylation to give a C3 exomethylene group, consistent with observations of other cephalosporin:SBL acyl-enzymes (36, 37, 38, 39).

### Ceftazidime binding induces large structural changes in KPC-2

**Figure 3:**
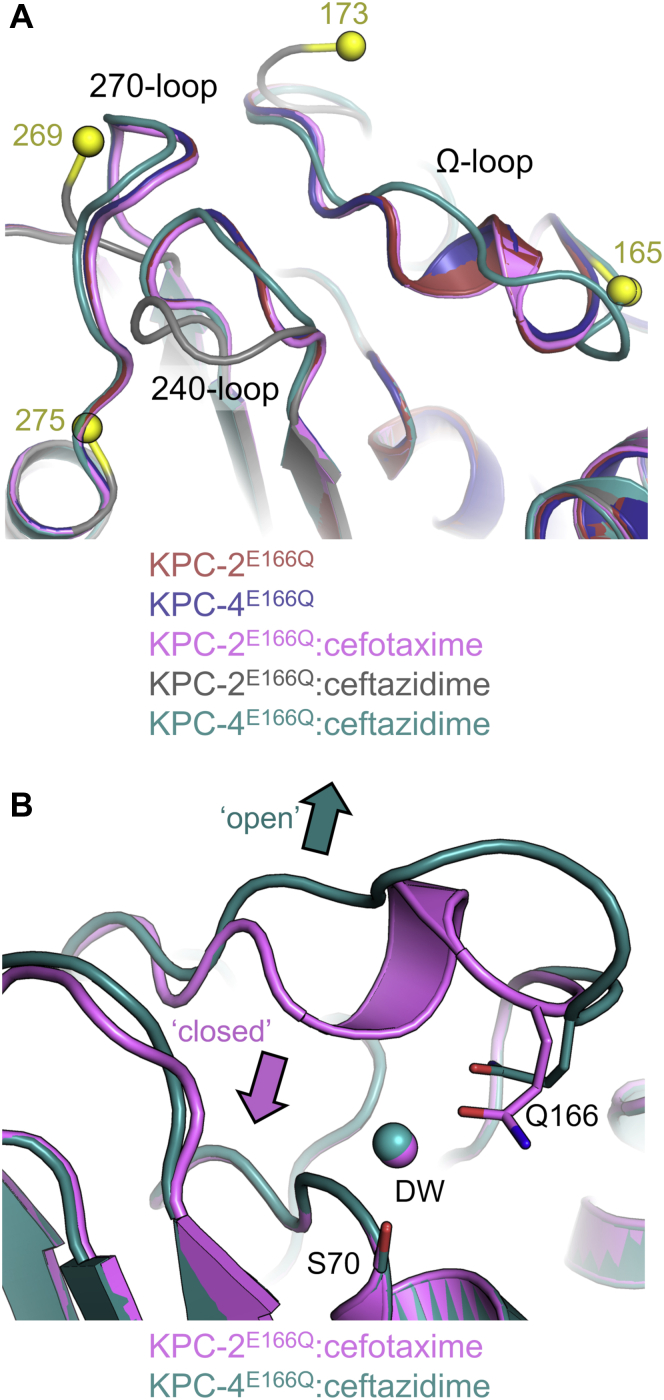
**Variable organisation of loops surrounding the active sites of KPC acyl-enzymes.***A*, conformational changes in the Ω, 240, and 270 loops (labeled) are manifest in superimpositions of KPC-2^E166Q^ (*red*), KPC-4^E166Q^ (*blue*), KPC-2^E166Q^:cefotaxime (*pink*), KPC-2^E166Q^:ceftazidime (*gray*), and KPC-4^E166Q^:ceftazidime (*teal*). The beginning and end of disordered regions in the KPC-2^E166Q^:ceftazidime crystal structures are represented as *yellow* spheres, and the corresponding residues numbered in the KPC-2^E166Q^ and KPC-4^E166Q^ structures. *B,* the open and closed conformations of the Ω loop in KPC^E166Q^ acyl–enzyme complexes, colored as in *A*. DW, water molecule in the putative deacylating position; KPC, *Klebsiella pneumoniae* carbapenemase.

On reaction with KPC-2^E166Q^, ceftazidime induces relatively large global changes (*C*_α_ RMSD = 0.618 Å compared with apo KPC-2^E166Q^; Table S3). In particular, the Ω loop (residues 165–170) and residues 270 to 274 (henceforth referred to as the 270 loop) are highly disordered, as evidenced by a lack of interpretable electron density (Figs. 3 and S3). Ω Loop restructuring appears necessary to prevent significant steric clashes of N170 with the aminothiazole ring of the C7 substituent (Fig. 2). Although residues 239 to 243 (the 240 loop) manifest well-defined experimental electron density (*B*-factor = 18.6 Å^2^), they move considerably, relative to their positions in the apoenzyme, that is, away from the Ω loop and toward the position of the 270 loop in uncomplexed KPC-2 (Figs. 2 and 3 and S2). The reasons for this substantial change in the position of the 240 loop are unclear, but could reflect the proximity of the flexible/disordered Ω loop, the binding mode of the ceftazidime aminothiazole ring, and/or the provision of hydrophobic interactions between V240 and the methyl groups of the ceftazidime C7 substituent (closest distance between CH_3_ groups is 4.5 Å). This movement of the 240 loop, and the consequent potential for steric clashes, can additionally explain the absence of electron density for the 270 loop.

**Figure 4:**
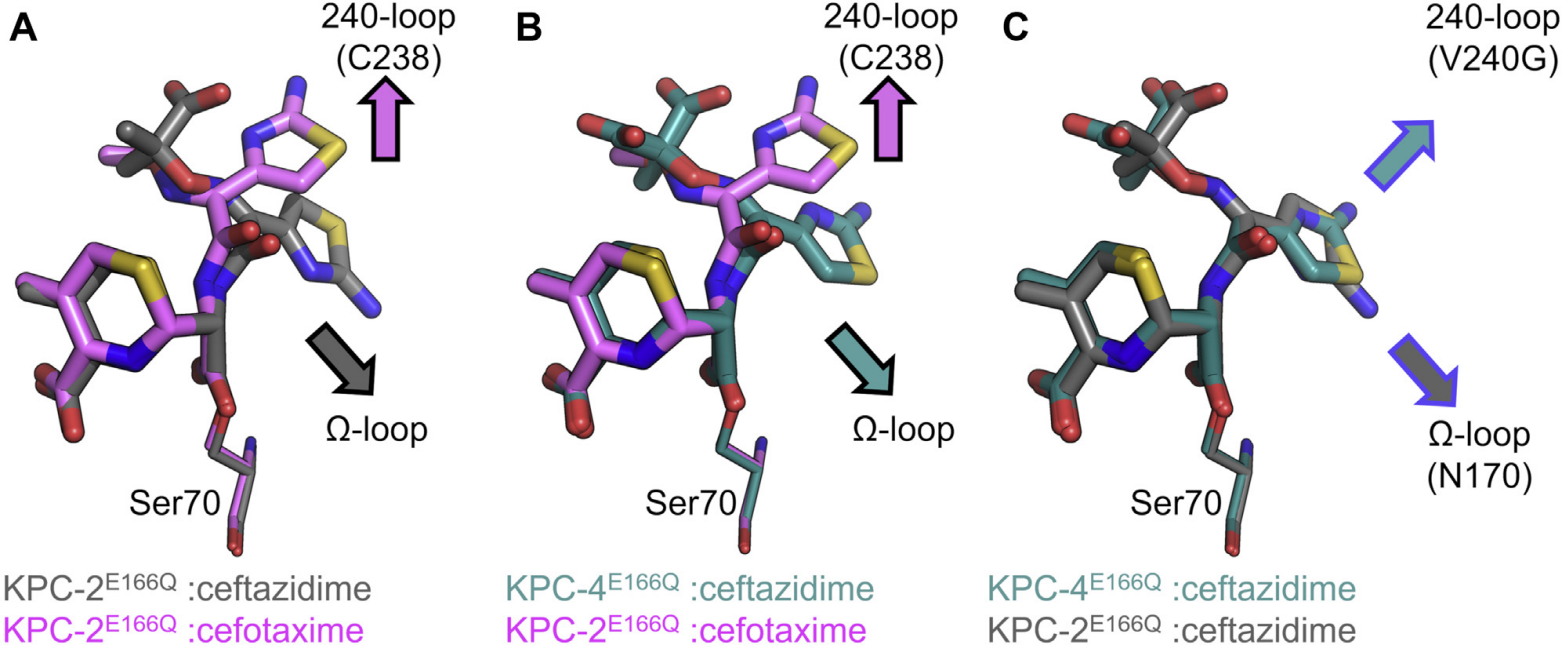
**Conformations of cefotaxime and ceftazidime in KPC acyl-enzymes.***A*, conf B of KPC-2^E166Q^:cefotaxime (*pink*) and KPC-2^E166Q^:ceftazidime (*gray*). *B*, conf B of KPC-2^E166Q^:cefotaxime (*pink*) and KPC-4^E166Q^:ceftazidime (*teal*). *C*, KPC-2^E166Q^:ceftazidime (*gray*) and KPC-4^E166Q^:ceftazidime (*teal*). *Arrows* with a *black* outline represent positioning of the aminothiazole ring of the C7 side chain. Arrows with a *blue* outline highlight the position of the amine group on the aminothiazole ring. KPC, *Klebsiella pneumoniae* carbapenemase.

Ceftazidime binding to KPC-4^E166Q^ (*i.e.* KPC-2^E166Q^ P104R/V240G) induces notably less pronounced conformational changes, with *C*_α_ RMSDs of 0.269/0.254 Å compared with unliganded KPC-4^E166Q^ or KPC-2^E166Q^, respectively (Table S3). Indeed, for the KPC-4^E166Q^: ceftazidime acyl-enzyme, the 240 loop (containing the V240G substitution) and the 270 loop are both well defined by the electron density (*B*-factors 15.78 and 20.0 Å^2^, respectively) and occupy similar positions to those in the unliganded KPC-4^E166Q^/KPC-2^E166Q^ and KPC-2/KPC-4 structure. However, although the complete Ω loop could be modeled in relatively weak electron density (all atom *B*-factors for residues 165–175 are more than twice those of the remainder of the main chain [43.79 *versus* 18 Å^2^]), a likely consequence of the amino group of the ceftazidime C7 aminothiazole ring being directed out of the active site (Figs. 2*B* and 4*C* and S3), it adopts an open conformation compared with its position in the unmodified enzyme (Fig. 3). Thus, structural changes in the Ω loop are required for ceftazidime to bind to either KPC-2^E166Q^ or KPC-4^E166Q^, consistent with the high *K*_M_ values for the native enzymes (530 and 640 μM; Table 1).

In contrast, cefotaxime binding to KPC-2^E166Q^ causes minimal structural changes compared with unmodified KPC-2^E166Q^ (*C*_α_ RMSD = 0.145 Å; Table S3). Furthermore, the Ω loop, 240 loop, and 270 loop are all well defined by the electron density (*B*-factors 28.8, 18.0, 27.9 Å^2^, respectively), and conserved active-site residues all occupy similar positions to those found in unmodified KPC-2 (33) and KPC-2^E166Q^, with the deacylating water situated between N170 and Q166 (Fig. 2*C* and S2). Thus, although we note that overall *K*_M_ values for ESOC turnover (Table 1) reflect contributions from the microscopic rate constants for multiple individual steps along the hydrolysis pathway (40), and that in consequence, *K*_M_ will be sensitive to changes in the structures and dynamics of transient species as well as those of the acyl–enzyme state observed here, our structural data therefore suggest that the lower *K*_M_ value for KPC-2–catalyzed cefotaxime, compared with ceftazidime, hydrolysis may in part reflect the lack of a requirement for significant active-site rearrangements in the cefotaxime acyl-enzyme.

### Interactions of the cephalosporin acyl-enzymes with the KPC active site

Ceftazidime and cefotaxime make several hydrogen bonds to the same active-site residues in KPC-2^E166Q^ and KPC-4^E166Q^ (Fig. 2), reflecting their similar core structures. In all three complexes, the oxyimino-cephalosporin C-8 (β-lactam) carbonyl oxygen binds in the oxyanion hole formed by the backbone amides of S70 and T237 (35) (2.7 and 2.9–3.0 Å, respectively), whereas the N5 (β-lactam) nitrogen hydrogen bonds with the S130 side chain (2.8 Å). The C-4 carboxylate makes weaker interactions with S130 (3.0–3.1 Å), and with the backbone oxygen of T216 *via* a water molecule (Wat2) but participates in stronger hydrogen bonds with the side chains of T235 and T237. In all three KPC complexes, the N132 side chain nitrogen points toward the C7 amide carbonyl oxygen (3.0–3.1 Å).

The three structures differ in the positioning of the aminothiazole ring of the C7 substituent (Fig. 4). In particular, the cefotaxime aminothiazole ring points away from the KPC-2 active site, toward C238 on the 240 loop, whereas in the ceftazidime acyl-enzymes, the ring is buried deeper within the active site (Figs. 2 and 4). In both KPC-2 and KPC-4, the ceftazidime aminothiazole ring is positioned where N170 typically resides in the apoenzyme (Fig. S2), explaining why the Ω loop is repositioned in both complexes (to the point that it could not be modeled in the KPC-2:ceftazidime complex; Fig. 3) and why in KPC-4 N170 points into bulk solvent, away from the active site, thus enlarging the active site to accommodate ceftazidime (Fig. 2*B*). These changes to the KPC-4 Ω loop likely cause Q166 to interact with a water molecule (WAT3) positioned 2.5 Å above the water in the putative deacylating position (DW, Fig. 2*B*). Furthermore, in KPC-2^E166Q^ the ceftazidime aminothiazole amino group points towards the position of N170 in unliganded KPC-2, whereas in KPC-4 the amino group instead points away from the Ω loop and towards the 240 loop, likely reflecting the reduced potential for steric clashes arising from the V240G substitution (Fig. 4*C*).

### The positions of P/R104 and W105 in KPC acyl-enzymes

**Figure 5:**
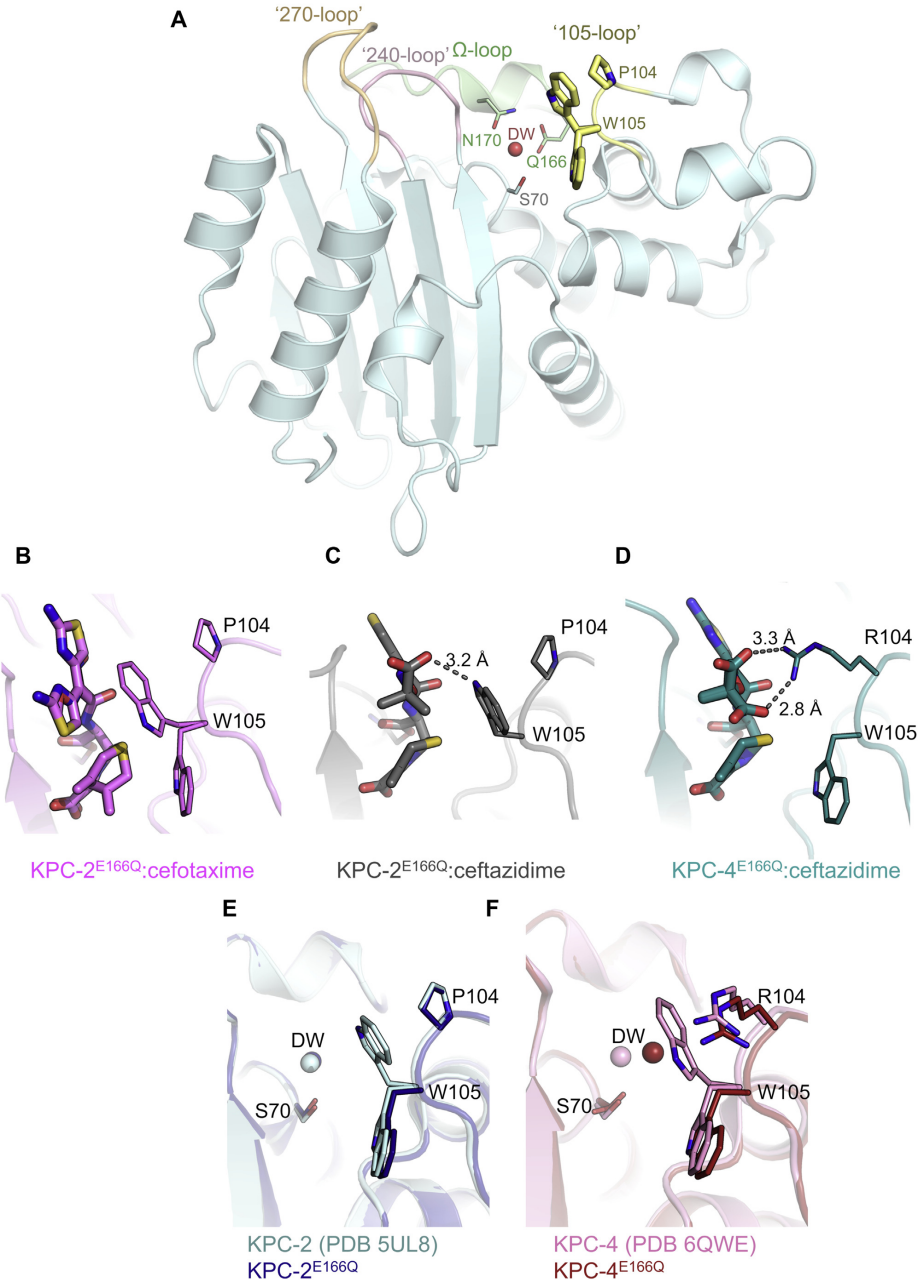
**Positioning of residues P/R104 and W105 in KPC crystal structures.** Cefotaxime/ceftazidime are represented as thick sticks. Residues 104 and 105 are shown as sticks and labeled. Distances of potential interactions between 104/105 are highlighted as gray dashes. *A*, representative view of the overall KPC-2 fold (PDB 5UL8), showing positions of key active-site residues and loops (labeled). *B*, KPC-2^E166Q^:cefotaxime (*pink*), *C,* KPC-2^E166Q^:ceftazidime (*gray*), *D*, KPC-4^E166Q^:ceftazidime (*teal*), *E*, unliganded KPC-2 (*pale blue*) and KPC-2^E166Q^ (*dark blue*), and *F*, unliganded KPC-4 (*pink*) and KPC-4^E166Q^ (*red*). Note the multiple conformations adopted by W105 in the various structures. DW, water molecule in the putative deacylating position; KPC, *Klebsiella pneumoniae* carbapenemase; PDB, Protein Data Bank.

Residues 104 and 105 (Fig. 5*A*), located adjacent to class A SBL active site, have been implicated in discrimination between substrates and stabilization of reaction intermediates, as supported by studies of residue 105 across a range of enzymes (TEM-1 (41), KPC-2 (42, 43), and SME-1 (44), among others (45, 46)). Here, we observe multiple conformations of W105 (Fig. 5), as previously described in structures of unliganded KPC enzymes from the *P*2_1_2_1_2 crystal form (31, 33). In the KPC-2^E166Q^:cefotaxime complex, W105 was modeled in two conformations (Fig. 5*B*; occupancies 0.53 and 0.47), similar to those observed in unliganded KPC-2 (33) (Fig. 5*E*). This could reflect the possibility for hydrophobic interactions between W105 and either the aminothiazole or dihydrothiazine rings. Conversely, in the KPC-2^E166Q^:ceftazidime complex, W105 adopts a single conformation that is well defined by the electron density, with the indole nitrogen hydrogen bonding to the ceftazidime C7 carboxylate (3.2 Å; Fig. 5*C*).

In KPC-4^E166Q^:ceftazidime (containing the P104R substitution), W105 adopts a conformation closer to that in apo KPC-4^E166Q^ (Fig. 5*F*) and KPC-2^E166Q^:cefotaxime (Fig. 5*B*), pointing away from the bulky R104 and into the active site (Fig. 5*D*), where it can make a hydrophobic interaction with the dihydrothiazine ring. R104 is directed toward ceftazidime, making hydrogen bonds with the ceftazidime C7 side chain carboxylate (conformation A = 2.8 Å and conformation B = 3.3 Å). The orientation of W105 within the KPC active site is therefore both substrate dependent and influenced by the P104R substitution in KPC-4.

### KPC-2 demonstrates increased Ω loop flexibility, compared with KPC-4, in MD simulations of the ceftazidime acyl-enzyme

To test the apparent conformational flexibility observed in our crystal structures, as evidenced in both the KPC-2^E166Q^:ceftazidime and KPC-4^E166Q^:ceftazidime acyl–enzyme complexes by structural rearrangements and/or the absence of experimental electron density, we undertook MD simulations of unliganded and acyl–enzyme forms of KPC-2 and KPC-4. For these simulations, Q166 was replaced with glutamate (E166) in the ceftazidime complexes to regenerate the active enzyme *in silico*, and disordered regions of the KPC-2^E166Q^:ceftazidime acyl-enzyme were manually built using restraints and the Modloop server (47) (minimized complex shown in Fig. S4*A*). To investigate the effect of the E166Q substitution on protein dynamics, we initially subjected the eight complex/unliganded structures (KPC-2, KPC-4, KPC-2^E166Q^, KPC-4^E166Q^, KPC-2:ceftazidime, KPC-4:ceftazidime, KPC-2^E166Q^:ceftazidime, and KPC-4^E166Q^:ceftazidime) to 100 ns MD simulations in triplicate. *C*_α_ RMSDs of all residues remained stable throughout 100 ns, and active-site residue RMSDs were similar between E166 and Q166 structures (Figs. S5 and S6). Therefore, as the Q166 and E166 structures behaved similarly, we focused on the wildtype (*i.e.* E166) enzymes in subsequent 500 ns simulations (repeated in triplicate).

**Figure 6:**
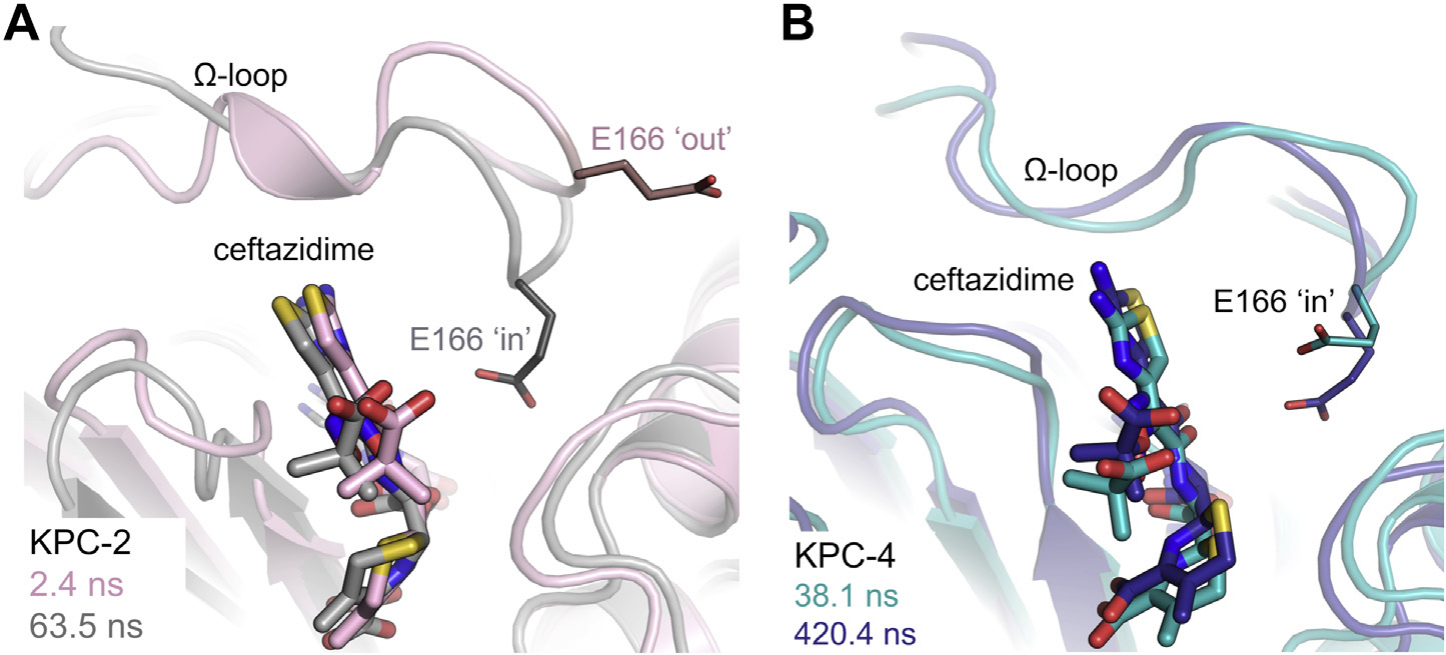
**Conformations of E166 in KPC-2 and KPC-4 molecular dynamics trajectories.***A*, snapshots of KPC-2:ceftazidime acyl-enzyme identified by cluster analysis, which represent "in" and "out" conformations of E166. 2.5 ns (*gray*) and 63.5 ns (*pale pink*). *B*, snapshots of KPC-4:ceftazidime acyl-enzyme identified by cluster analysis, which represent movements of E166 pointing "in" to the active site. 38.1 ns (*teal*) and 420.4 ns (*blue*). In KPC-4:ceftazidime simulations, E166 only samples "in" conformations. KPC, *Klebsiella pneumoniae* carbapenemase.

Backbone (*C*_α_) RMSD values remained stable throughout 500 ns (Fig. S7), with unliganded KPC-2 and KPC-4 overall marginally more rigid than their respective ceftazidime acyl-enzymes. By-residue root mean square fluctuation (RMSF) analysis showed the Ω, 240, and 270 loops of both the KPC-2 and KPC-4:ceftazidime acyl–enzyme complexes to be more mobile than the remainder of the protein, but, notably, this enhanced mobility was not evident in unliganded KPC-2 or KPC-4 (Fig. S8). Of the four systems studied, the KPC-2:ceftazidime acyl-enzyme exhibited consistently higher RMSF values across all three loops, reflecting the weak or absent crystallographic electron density for these regions (Figs. 2 and 3 and S3 and S8). Flexibility of the Ω loop is manifest within 1 ns of MD equilibration in simulations of both the KPC-2 and KPC-4 ceftazidime acyl-enzymes, and is accompanied by movements of the ceftazidime C7 side chain (Fig. S4). Visual inspection of the complete MD trajectories revealed E166 (and the entire Ω loop) to be highly mobile in simulations of both the KPC-2 and KPC-4 ceftazidime acyl-enzymes, consistent with weak (KPC-4) or absent (KPC-2) experimental electron density for this region. In particular, in the KPC-2 acyl-enzyme, E166 adopted two distinct orientations (Fig. 6), either pointing “in” to the active site or “out” into solvent where it is unable to interact with the deacylating water molecule (DW) or with other components of the active-site. In KPC-4, E166 adopted only the “in” conformation, although the Ω loop still underwent movements during the simulations.

To identify favored conformations of ceftazidime, and associated movements of the Ω loop, MD trajectories of KPC-2:ceftazidime were investigated with cluster analysis (48, 49). This identified distinct clusters relating to the “in” or “out” positions of E166 and the orientation of the ceftazidime C7 aminothiazole ring (Fig. S9). Three clusters are presented in Figure S9. In the first, E166 points in (Fig. S9*B*), and the C7 aminothiazole of bound ceftazidime is oriented as in the crystal structure. In the second, the Ω loop and E166 move out and away from the active site, but ceftazidime retains the orientation observed in the first cluster (Fig. S9*C*). In the third, there is a substantial change in the ceftazidime binding mode as the aminothiazole ring occupies the space created by the movement of the Ω loop that positions E166 in the “out” conformation (Fig. S9*D*). To accompany these conformational changes, P104 moves closer (from ∼6.7 Å after 2.5 ns to ∼3.5 Å after 422 ns of simulation) to the methyl groups of the C7 oxyimino group, with which it can then participate in van der Waals interactions. Despite these differences, the acyl-enzyme β-lactam–derived C8 carbonyl remains within the oxyanion hole for the entire simulation (Fig. S10); this contrasts with previous studies of class A enzymes with carbapenems that show that the carbapenem carbonyl can flip out of the oxyanion hole (50, 51, 52). Equivalent cluster analysis of MD trajectories for the KPC-4:ceftazidime complex reveals that E166 is directed into the active site in all clusters (Fig. S11), with the ceftazidime aminothiazole oriented as in the crystal structure and the acyl–enzyme carbonyl remaining in the oxyanion hole. There are small movements of the R104 side chain, which in the crystal structure hydrogen bonds to the C7 oxyimino group carboxylate; this interaction between R104 and the carboxylate (Fig. S12) is present in most simulations. Movements of R104 during MD may therefore correlate with movements of this C7 group, which throughout remains at hydrogen-bonding distance (∼2.7–3.0 Å) to the arginine Nε and Nη atoms (Figs. S11 and S12).

To quantify how often E166 points in or out of the active site, the distance between the E166 C_δ_ and the *C*_α_ of the nucleophilic S70 was monitored across all simulations. In unliganded KPC-2 and KPC-4, the Ω loop remains stable, with E166 directed into the active site in 100% of frames (Fig. S13), and the distance between E166 and S70 remaining below 7 Å (averaging 5.04 Å and 5.14 Å for KPC-2 and KPC-4, respectively). In the KPC-4:ceftazidime acyl-enzyme, the average E166 to S70 distance was 9.5 Å, with E166 pointing toward S70 in ∼100% of frames, reflecting the conformational change of the Ω loop in the crystal structure into a more open state (Figs. 3 and 6 and S14), similar to that in a CTX-M-14:ceftazidime crystal structure (PDB 5TW6 (36)). In contrast, in trajectories of the KPC-2:ceftazidime acyl-enzyme, E166 is on average 15.17 Å from S70, with this distance distributed between 12 and 18.6 Å in ∼98% of frames, *i.e.*, E166 is predominantly directed out of the active site. E166 is only within 11 Å of S70, and thus oriented in the in conformation observed for KPC-4, in frames comprising <3% of the trajectory (Fig. S14*C*).

## Discussion

The ESOC ceftazidime is a widely used antibiotic and relatively poor substrate for the globally distributed KPC carbapenemases. In recent years, KPC variants have emerged with increased ceftazidime turnover. In particular, acquisition of two substitutions in KPC-4, V240G, and P104R, results in a 40-/50-fold increase in catalytic efficiency over the KPC-2 parent (13), leading in producer strains to an increase in minimal inhibitory concentration for ceftazidime (12, 13). Our steady-state kinetic data show this to be due to increased turnover (*k*_*cat*_) rather than changes in *K*_M_. Using deacylation-deficient mutants, we captured high-resolution crystal structures of the KPC-2 and KPC-4 ceftazidime acyl-enzymes, alongside the equivalent complex of KPC-2 with cefotaxime, an ESOC with a smaller C7 side chain that is a better substrate. The combination of these structures with MD simulations presents an explanation for enhanced ceftazidime turnover by KPC-4. Such information is of growing clinical relevance with the emergence of further KPC variants and the likely increase in ceftazidime use (and selection pressure), arising from clinical introduction of the ceftazidime–avibactam combination (26).

KPC-4 turns over ceftazidime an order of magnitude more efficiently than KPC-2 (*k*_cat_/*K*_M_ values: 0.13 μM^−1^ s^−1^ and 0.0035 μM^−1^ s^−1^, respectively). Our crystal structure of the KPC-4:ceftazidime complex shows that both the P104R and V240G substitutions affect ceftazidime binding and hydrolysis: the former directly through interaction of R104 with the ceftazidime C7 carboxylate and indirectly by dictating positioning of W105; the latter by enabling accommodation of the aminothiazole ring with its amino substituent pointing away from the Ω loop, maintaining E166 in an orientation compatible with productive deacylation. Thus, although ceftazidime binding to KPC-2 induces conformational changes in the 240, 270, and Ω loops, binding to KPC-4 did not alter positioning of the 240 and 270 loops and involved less pronounced changes to the Ω loop than in KPC-2. The conclusion that ceftazidime binding is more disruptive to KPC-2 is supported by MD simulations of the acyl-enzymes, which show the KPC-2 Ω loop to be more mobile and with E166 generally oriented away from S70, in a conformation incompatible with its role activating the deacylating water. We conclude that the KPC-2:ceftazidime acyl-enzyme predominantly adopts a conformation incompatible with E166-catalyzed hydrolysis, whereas the KPC-4 acyl–enzyme conformation, specifically that of the Ω loop, remains permissive for deacylation, with E166 and S70 consistently remaining in proximity. These findings are consistent with the respective turnover numbers (ceftazidime *k*_cat_ values: 1.9 s^−1^ and 81 s^−1^ for KPC-2 and KPC-4, respectively), and the crystal structure of the KPC-2:cefotaxime acyl-enzyme, where, in contrast to the above, the conformations of the 240, 270, and Ω loops are little affected by binding of this relatively good substrate (*k*_cat_ = 76 s^−1^ and *K*_M_ = 200 μM). This change in the respective populations of the "in" and "out" conformations of E166 can account for the difference in activity toward ceftazidime between KPC-2 and KPC-4.

Differences between the two KPC variants are less apparent in MD simulations of the uncomplexed (apo) enzymes, with increased mobility of the Ω loop only evident after ceftazidime acylation. Furthermore, comparison with others' MD data suggests that different β-lactams cause different effects upon KPC dynamics after acylation: the active-site loop bearing W105, but not the 240, 270, or Ω loops, exhibits increased mobility in the complex with the penam sulfone inhibitor PSR-3-226 (53). W105 has also been identified as governing transitions between catalytically permissive (interacting with the oxyanion hole) and nonpermissive (loss of oxyanion hole interactions) conformations in simulations based upon models (*i.e.* docked rather than crystallographically defined) of the KPC-2:meropenem acyl-enzyme (52).

Our acyl–enzyme structures can also explain why various KPC point variants, several of which are associated with clinical failure of the ceftazidime:avibactam combination, display altered activity toward ceftazidime. Substitutions characterized in ceftazidime:avibactam-resistant strains include H274Y (KPC-3 (13)); H274Y combined with deletion of residues 242 and 243 (KPC-28 (30)); V240G (28); T243M/A (28, 29); and D179Y/N (28, 54, 55, 56). These mutations (Fig. S15) are all within loops whose conformations are significantly affected by ceftazidime binding, where repositioning of the 240 loop necessitates conformational changes in the 270 loop to avoid steric clashes with the ceftazidime aminothiazole ring. From our results, deletions or substitutions in the 240 loop are expected to reduce such clashes, specifically by better accommodating the ceftazidime aminothiazole ring. In this context, we note that deletions in the 240 loop (KPC-28) substantially lower ceftazidime *K*_M_ values compared with both KPC-2 and KPC-3. The H274Y substitution may further reduce the energetic burden associated with 240 loop reorganization. Substitutions, such as D179Y, in and around the Ω loop (which we show to be highly mobile in both crystal structures and MD simulations of the ceftazidime acyl-enzyme), probably aid ceftazidime acylation by modifying Ω loop behavior and dynamics to favor acylation-competent conformations of E166. The D179Y mutation may also affect the salt bridge between D179 and R164 (a residue itself shown to be important to ceftazidime turnover (17)).

Our results make possible comparisons of the mechanisms by which different class A β-lactamases expand their activity spectrum to encompass ESOCs, specifically ceftazidime. The basis for ESOC hydrolysis by ESBLs has been extensively studied in the TEM, SHV, and CTX-M SBL families, where efficient turnover requires acquisition of point mutations by a parent enzyme that lacks such activity (19, 57). In the TEM family, the parent TEM-1 enzyme exhibits poor cefotaxime or ceftazidime turnover and variants, such as R164S (19, 58), E104K (19), or E240K (58) (also associated with activity against ceftazidime in SHV enzymes (20)) enhance ceftazidime turnover. The first of these is proposed to increase conformational heterogeneity of the Ω loop to better accommodate ceftazidime (59), whereas E104K/E240K may participate in direct interactions with ceftazidime (58). In the CTX-M ESBL family, where the parent hydrolyzes cefotaxime efficiently but ceftazidime poorly, turnover of the latter is moderately improved by point mutations such as P167S or D240G. These are proposed to increase Ω loop flexibility to access a more open conformation, improving accommodation of ceftazidime, while E166 remains in a deacylation-competent orientation (60). This contrasts with KPC, in which our high-resolution crystal structures and MD simulations indicate that greatly enhanced ceftazidime turnover by KPC-4 (compared with KPC-2) is instead associated with *reduced* flexibility, or increased stability, of the acyl-enzyme Ω loop, constraining E166 in an orientation compatible with productive deacylation. This is further highlighted by the KPC-2:cefotaxime acyl–enzyme crystal structure in which the Ω loop is highly stable and close to the unliganded conformation, rationalizing better cefotaxime turnover *in vitro*.

Collectively, the results support the hypothesis that extension of activity toward expanded-spectrum cephalosporins, specifically ceftazidime, through acquisition of point variants by different class A β-lactamases, is associated with changes in Ω loop dynamics and the sampling of acyl–enzyme conformations that increase populations of deacylation-competent states. However, despite the structural similarity between class A β-lactamases, available data indicate that populated acyl–enzyme conformations, and their associated hydrolytic activities, vary between different SBLs. This is exemplified by the apparently opposing effects of increased Ω loop flexibility upon ceftazidime hydrolysis in the CTX-M (36, 60) and KPC enzyme families. These differences likely reflect differing active-site structures and associated activity spectra of the progenitor enzymes as well as differences in stability/dynamics of the overall enzyme structure. Our results reveal that studies of acyl-enzymes and their reactivity are particularly important for understanding deacylation activity (61). Molecular simulations have an important role to play in characterizing the dynamics and activity of acyl-enzymes and can potentially be used as computational assays of activity (62). For example, likely activity would be indicated for acyl-enzymes of KPC variants where simulations show E166 predominantly in “in” conformations, whereas lower deacylation activity would be predicted from simulations showing E166 further from the active site. Extension of simulations to the quantum mechanics/molecular mechanics (QM/MM) level could then enable a quantitative correlation between populations of different conformations and turnover rates.

In conclusion, our determination of the first crystal structures of acyl-enzymes for the clinically important KPC enzyme identifies how two point substitutions (P104R/V240G) dramatically improve ceftazidime hydrolysis. Our data demonstrate both the importance of Ω loop dynamics in turnover of complex ESOC substrates, and the existence of a trade-off between the Ω loop flexibility necessary to accommodate such antibiotics and the cost to catalytic efficiency of the associated disruption to the active site, specifically with respect to the orientation of the E166 general base and its ability to participate in deacylation. Point mutations acquired by KPC-4 resolve this conflict by modifying the conformational distribution of the ceftazidime acyl-enzyme to favor the deacylation-competent orientation of E166. Our findings therefore show how the spectrum of activity of KPC enzymes is modified by variation and suggest that future cephalosporin development can be aided by understanding conformational dynamics during catalysis and taking into account enzyme–substrate interactions involved in the evolution of resistance. The approach described here should aid activity prediction for variant enzymes, whereas the inactive acyl–enzyme conformers that we identify can potentially be targeted in β-lactam development. Finally, our finding that a naturally selected KPC variant alters specificity by affecting the structure and dynamics of the acyl-enzyme, rather than the ground (unliganded) state, highlights the need to consider the effects on reaction intermediates in studies of the evolution of β-lactamase activity.

## Experimental procedures

### Site-directed mutagenesis of the KPC variants

DNA encoding for KPC-2 and KPC-4 codons 25 to 293 were cloned into pET28a (Noavagen). Deacylation-deficient (E166Q) mutants were generated using primers 5′-TCA GCT CCA GCT GCC AGC GGT CCA G-3′ and 5′-CTG GAC CGC TGG CAG CTG GAG CTG A-3′ and the QuikChange II XL lightening SiteDirected Mutagenesis Kit, according to the manufacurer's instructions (Agilent Genomics).

### Protein crystallization, antibiotic soaking, and data collection

The KPC variants (KPC-2, KPC-4, KPC-2^E166Q^, and KPC-4^E166Q^) were purified and crystallized as described previously (31). For unliganded data sets of KPC-2^E166Q^ and KPC-4^E166Q^, crystals were soaked in mother liquor supplemented with 25% glycerol before cryocooling in liquid nitrogen. KPC-2^E166Q^ crystals and KPC-4^E166Q^ crystals were soaked in solutions of 30 mM ceftazidime (Merck) and KPC-2^E166Q^ crystals in 20 mM cefotaxime (Merck). Antibiotic solutions were made in mother liquor supplemented with 20% to 30% glycerol. Crystals were soaked from 5 min to several hours; the best data sets were collected at 3.5 h (KPC-2^E166Q^:ceftazidime), 2.5 h (KPC-2^E166Q^:cefotaxime), and 30 min (KPC-4^E166Q^:ceftazidime). Diffraction data were collected at Diamond Light Source beamlines I03, I04, and I24 and ALBA beamline BL13 XALOC. Images were indexed and integrated using Dials (63) in the Xia2 pipeline (64) (Diamond Light Source data sets) or XDS (65) (ALBA data) and subsequently scaled in AIMLESS (CCP4 suite (66)). Crystallographic phases were calculated in Phaser (67) using PDB:5UL8 (33) (crystal structure of KPC-2) as a molecular replacement solution. Initial refinements in REFMAC 5 confirmed *F*_o_–*F*_c_ electron density consistent with bound ligand, before further rounds of refinement in phenix.refine (68) and manual model building in Coot (69). Geometry restrains for antibiotics were calculated using eLBOW in PHENIX, and omit maps were generated in PHENIX (68) from the final model in the absence of the antibiotic. Ligand occupancies were manually assigned based upon inspection of electron density and subsequently refined in Phenix with at least 10 rounds of refinement. Figures were generated in Pymol (www.pymol.org) .

### Enzyme assays

All enzyme assays were performed at 25 °C in 10 mM HEPES, pH 7.5, 150 mM NaCl in Greiner half area 96-well plates, and a Tecan Infinite 200 pro microplate reader. Steady-state kinetic parameters were calculated by measuring β-lactam antibiotic hydrolysis (cefotaxime Δε_262_ = −7660 (17), ceftazidime Δε_265_ = −7445, and nitrocefin Δε_486_ = 20,500 (31)). Initial rates (*V*_0_) of β-lactam hydrolysis were plotted against concentration of antibiotic; kinetic parameters were calculated and analyzed using the Michaelis–Menten curve in GraphPad Prism 6 (GraphPad Software, La Jolla, CA, USA; www.graphpad.com).

### MD simulations

The crystal structures of KPC-2 (PDB 5UL8), KPC-4 (PDB 6QWE), KPC-2^E166Q^, KPC-4 E^166Q^, KPC-2^E166Q^:ceftazidime, and KPC-4^E166Q^:ceftazidime were used as starting structures for molecular simulation. The unresolved regions of KPC-2^E166Q^:ceftazidime (because of poorly defined electron density) were built in to the sparse electron density using both KPC-4^E166Q^:ceftazidime and KPC-2^E166Q^:cefotaxime crystal structures as a reference. MODELLER (ModLoop (47)) was then used to predict loop conformations of the 165 to 170 and the 270 to 274 regions. The resulting KPC-2^E166Q^:ceftazidime structure and KPC-4^E166Q^:ceftazidime were used to model the unmodified enzyme by mutating residues Q166 back to wildtype E166 in WinCoot using the experimental electron density. The resulting eight protein crystal structures (KPC-2, KPC-4, KPC-2^E166Q^, KPC-4 E^166Q^, KPC-2^E166Q^:ceftazidime, KPC-4^E166Q^:ceftazidime, KPC-2:ceftazidime, and KPC-4:ceftazidime) were parameterized for molecular simulation. All crystallographically observed water molecules were included in the structures. Protonation states of titratable residues were determined using the PropKA 3.1 server. In tleap (AMBER16 (48)), hydrogens were added, and the systems were solvated using a 10 Å water box (TIP4P) with overall charges neutralized by addition of Na+ or Cl− atoms replacing bulk water molecules. The ceftazidime acyl–enzyme parameters were generated using the restrained electrostatic potential fitting as implemented in the RED Server (70). All structures underwent a standard minimization (600 of deepest descent and 600 steps of conjugate gradient), heating (25 to 298 K in 20 ps), and equilibration MM MD (1 ns) protocol. The structures were then simulated using MM MD in the AMBER16 simulation package (48) using the ff14SB (71) MM force field for protein, the TIP4P-Ew water model, and the General AMBER Force Field (48). All eight protein systems were simulated in triplicate runs for 100 ns, and KPC-2, KPC-4, KPC-2:ceftazidime, and KPC-4:ceftazidime were further simulated in triplicate runs of 500 ns. RMSD, RMSF, clustering, and distance analyses were performed in CPPTRAJ in AMBER16 (48). RMSD calculations were performed using the first frame (1 ps) as the reference.

## Data availability

For all crystal structures presented herein, coordinates and structure factors have been deposited to the Worldwide PDB under accession codes 6Z21, 6Z22, 6Z23, 6Z24, and 6Z25. All other relevant data are within the article.

## Conflict of interest

The authors declare that they have no conflicts of interest with the contents of this article.
