# Supplementary material for: Natural variants modify Klebsiella pneumoniae carbapenemase (KPC) acyl–enzyme conformational dynamics to extend antibiotic resistance
Source: J Biol Chem. 2020 Dec 3;296:100126. doi: 10.1074/jbc.RA120.016461 (PMC7949053; doi:10.1074/jbc.RA120.016461)
Supplement: Figures and Tables [file mmc1.pdf]

Supporting information for: **Natural variants modify Klebsiella pneumoniae carbapenemase (KPC) acyl-enzyme conformational dynamics to extend antibiotic resistance**

Catherine L. Tooke, Philip Hinchliffe, Robert A. Bonomo, Christopher J. Schofield, Adrian J. Mulholland, and James Spencer.

Supporting figures

Figure S1. Electron density maps of cefotaxime and ceftazidime acyl-enzymes of deacylation-deficient mutants of KPC-2 and KPC-4.

Figure S2. Comparisons of apo (unliganded) KPC-2^{E166Q} and KPC-4^{E166Q} structures with apo KPC-2 and KPC-4.

Figure S3. Overall views of oxyimino-cephalosporin acylenzyme crystal structures of KPC-2^{E166Q} and KPC-4^{E166Q}.

Figure S4. Minimized and equilibrated structures of KPC-2 and KPC-4 ceftazidime acyl-enzymes.

Figure S5. Backbone (C_α) RMSD for MD simulations of wild-type and E166Q KPC over 100 ns trajectories.

Figure S6. Average RMSD for active site residues and backbone C_α of wild-type and E166Q KPC over 100 ns trajectories.

Figure S7. Backbone (C_α) RMSD for MD simulations of KPC over 500 ns trajectories.

Figure S8. Average per residue C_α RMSF over 500 ns MD simulations.

Figure S9. KPC-2:ceftazidime acylenzyme conformations during molecular dynamics simulations identified by cluster analysis.

Figure S10. Positioning of the acyl-enzyme carbonyl in the oxyanion hole.

Figure S11. KPC-4:ceftazidime acylenzyme conformations during molecular dynamics simulations identified by cluster analysis.

Figure S12. Interaction between R104 and the ceftazidime C7' carboxylate in the KPC-4:ceftazidime acylenzyme.

Figure S13. Distribution of distances between E166 and S70 in unliganded KPC-2 and KPC-4.

Figure S14. Ω-Loop conformations during MD simulations of KPC-2 and KPC-4 ceftazidime acyl-enzymes.

Figure S15. KPC variant positions associated with ceftazidime:avibactam resistance.

Supporting tables

Table S1. Uniprot and GenBank identification numbers for KPC variants.

Table S2. Ligand occupancies, RSCC and B-factor

Table S3. C_α RMSDs for KPC crystal structures.

Table S4. Distances (Å) of potential H-bond interactions within unliganded KPC-2^{E166Q} and KPC-4^{E166Q}.

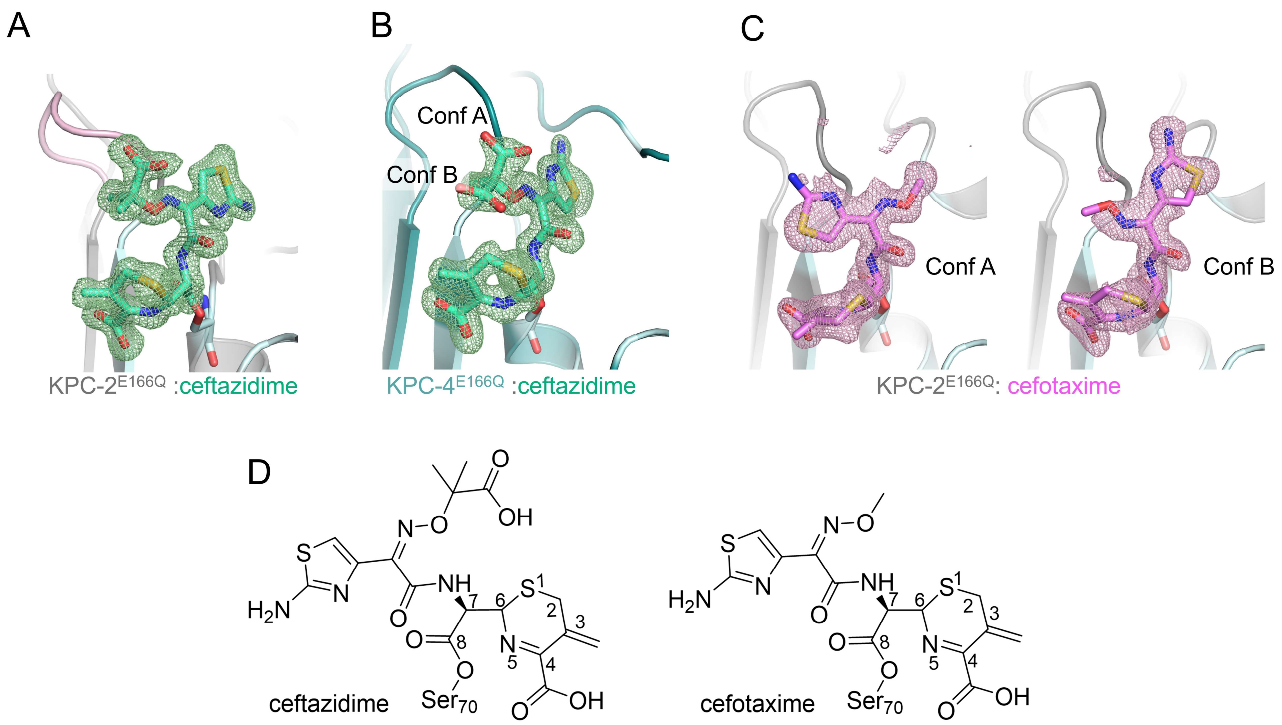

Figure S1. Electron density maps of cefotaxime and ceftazidime acyl-enzyme complexes of deacylation-deficient mutants of KPC-2 and KPC-4. Unbiased F_o-F_c electron density maps were calculated with the antibiotic derived atoms removed and are shown contoured at 3σ (pink or green mesh for cefotaxime or ceftazidime, respectively). The cefotaxime acyl-enzyme is represented as pink sticks and that of ceftazidime as cyan sticks. **(A)** KPC-2^{E166Q}:cefotaxime. **(B)** KPC-2^{E166Q}:ceftazidime. **(C)** KPC-4^{E166Q}:ceftazidime and **(D)** chemical representation of ceftazidime and cefotaxime acylenzymes. Ligand occupancies were manually assigned based upon inspection of electron density and subsequently refined in Phenix with at least 10 rounds of refinement.

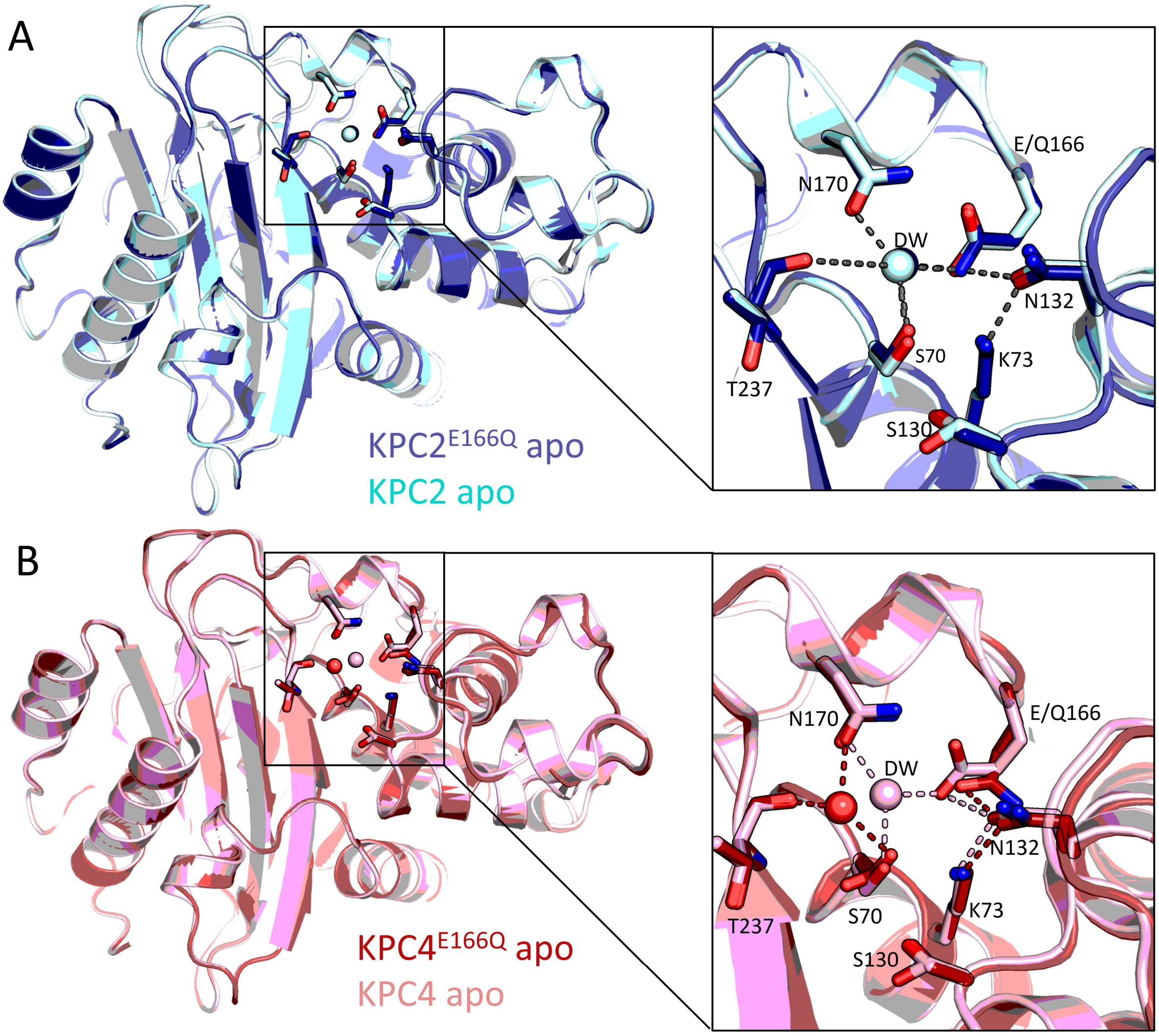

Figure S2. Comparisons of apo (unliganded) KPC-2^{E166Q} and KPC-4^{E166Q} structures with apo KPC-2 and KPC-4. Overall views of the protein fold are on shown on the *left*, with close up views from the active shown on the *right*. Key residues are highlighted as sticks, and the water molecules in the putative deacylating position as spheres (labelled DW), possible hydrogen bonding interactions are highlighted as dashes. **(A)** KPC-2^{E166Q} (dark blue) superimposed on KPC-2 (PDB:5UL8, pale blue) RMSD= 0.155 (Table S4). **(B)** KPC-4^{E166Q} (red) superimposed on KPC-4 (PDB:6QWE, pink) RMSD= 0.185 (Table S4).

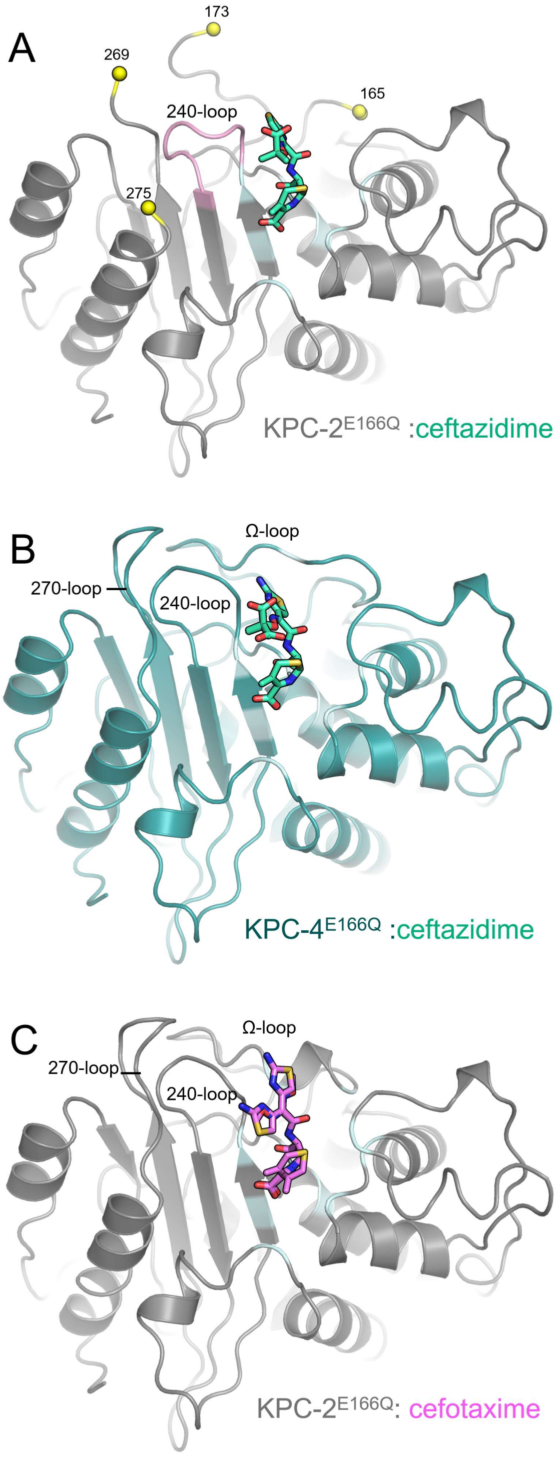

Figure S3. Overall views of oxyimino-cephalosporin acyl-enzyme complex crystal structures of KPC-2^{E166Q} and KPC-4^{E166Q}. The secondary structure is displayed as a cartoon; the cefotaxime acyl-enzyme is represented as pink sticks and that of ceftazidime as green cyan sticks. Positions of key active site amino acids residues are highlighted in cyan. **(A)** The KPC-2^{E166Q} protein backbone is colored gray. Regions which were not modelled due to incomplete or poor electron density are flanked by yellow spheres which represent the C_{α}s of the last residue modelled. Movement of the 240-loop is highlighted in pale pink. **(B)** The KPC-4^{E166Q} protein backbone is colored in teal. **(C)** KPC-2^{E166Q} is colored as in *A*.

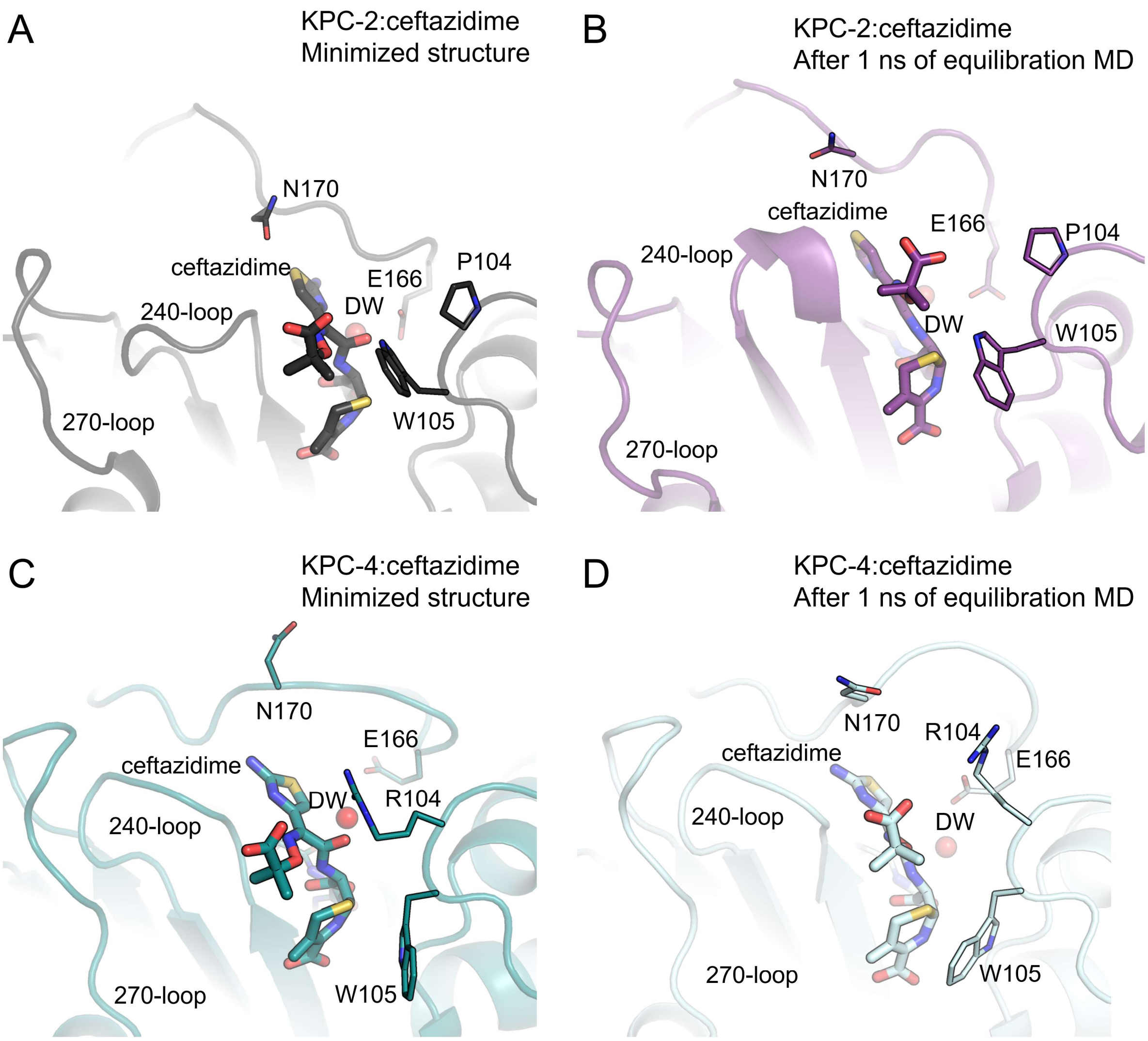

Figure S4. Minimized and equilibrated structures of KPC-2 and KPC-4 ceftazidime acyl-enzymes. (A) KPC-2:ceftazidime (gray) with modelled Ω-loop following minimization steps (see Methods). **(B)** KPC-2:ceftazidime following minimization, heating steps and 1 ns of equilibration MD (purple). **(C)** KPC-4:ceftazidime following minimization steps (teal). **(D)** KPC-4:ceftazidime following minimization, heating steps and 1 ns of equilibration MD (pale blue). The acylated S70, adjoining main chain of Cys69 and Ser71, and side chains of Glu166 and Asn170, Pro/Arg104 and Trp105 are presented as thin sticks.

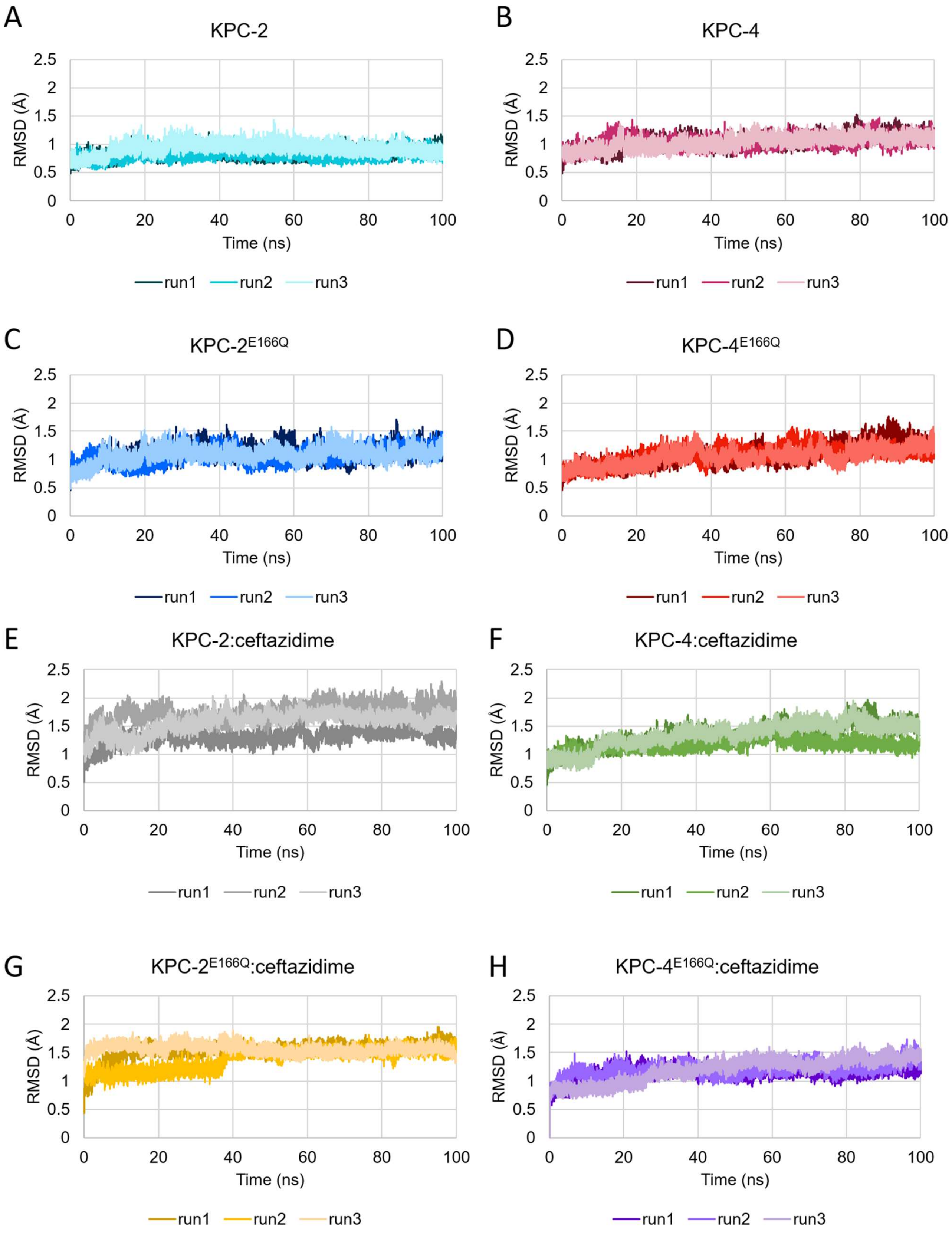

Figure S5. Backbone (C_α) RMSD for MD simulations of wild-type and E166Q KPC over 100 ns trajectories. Molecular dynamics simulations were run in triplicate for each enzyme (runs 1-3). **(A)** KPC-2, **(B)** KPC-4, **(C)** KPC-2^{E166Q}, **(D)** KPC-4^{E166Q} **(E)** KPC-2:ceftazidime, **(F)** KPC-4:ceftazidime, **(G)** KPC-2^{E166Q}:ceftazidime and **(H)** KPC-4^{E166Q}: ceftazidime.

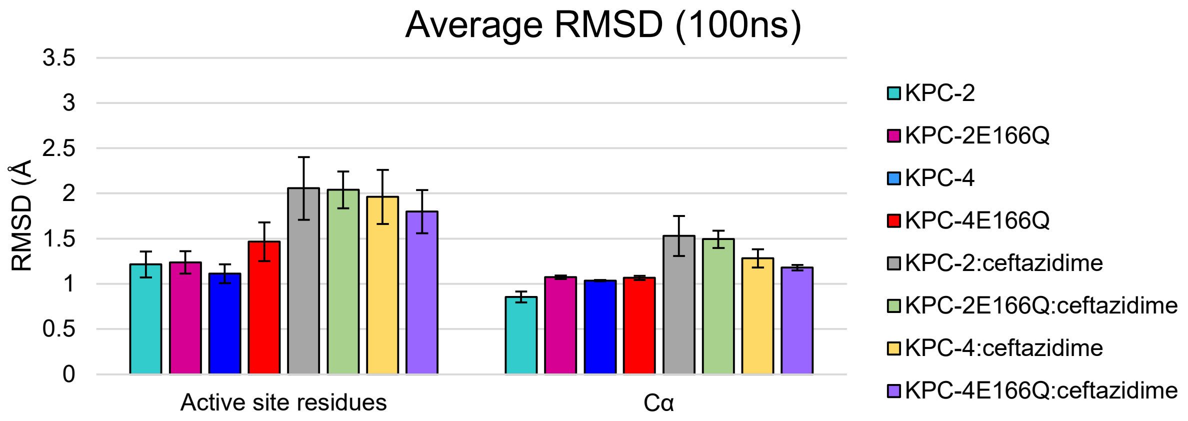

Figure S6. Average RMSD for active site residues and backbone C_α of wild-type and E166Q KPC over 100 ns trajectories. Averages of triplicate simulations with error bars representing the standard deviation. Active site residues include all the atoms of S70, K73, S130, T216, T235, T237, N170 and E166.

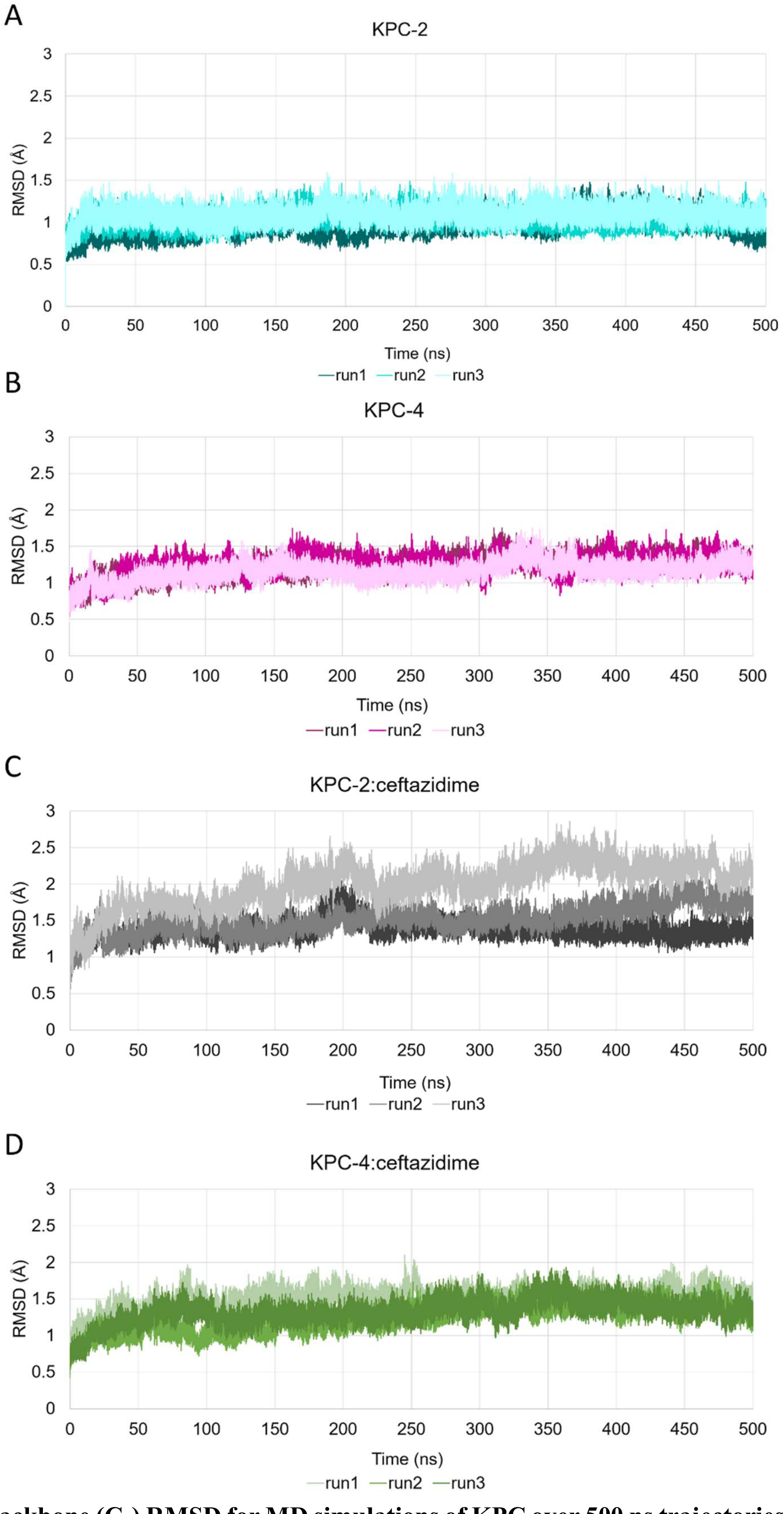

Figure S7. Backbone (C_α) RMSD for MD simulations of KPC over 500 ns trajectories. Simulations were run in triplicate for each enzyme (runs 1-3). **(A)** KPC-2, **(B)** KPC-4, **(C)** KPC-2:ceftazidime and **(D)** KPC-4:ceftazidime.

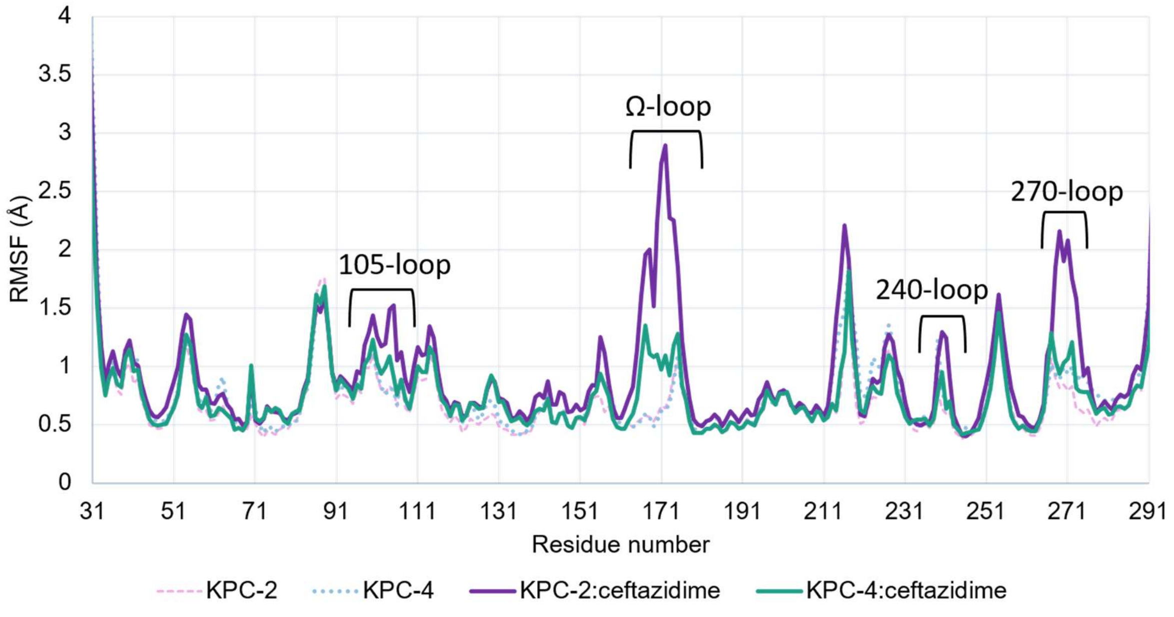

Figure S8. Average per residue C_α RMSF over 500 ns MD simulations. KPC-2 (dashed pink line), KPC-4 (dotted blue line), KPC-2:ceftazidime (solid purple line) and KPC-4:ceftazidime (solid teal line). In each case the average of triplicate runs is shown.

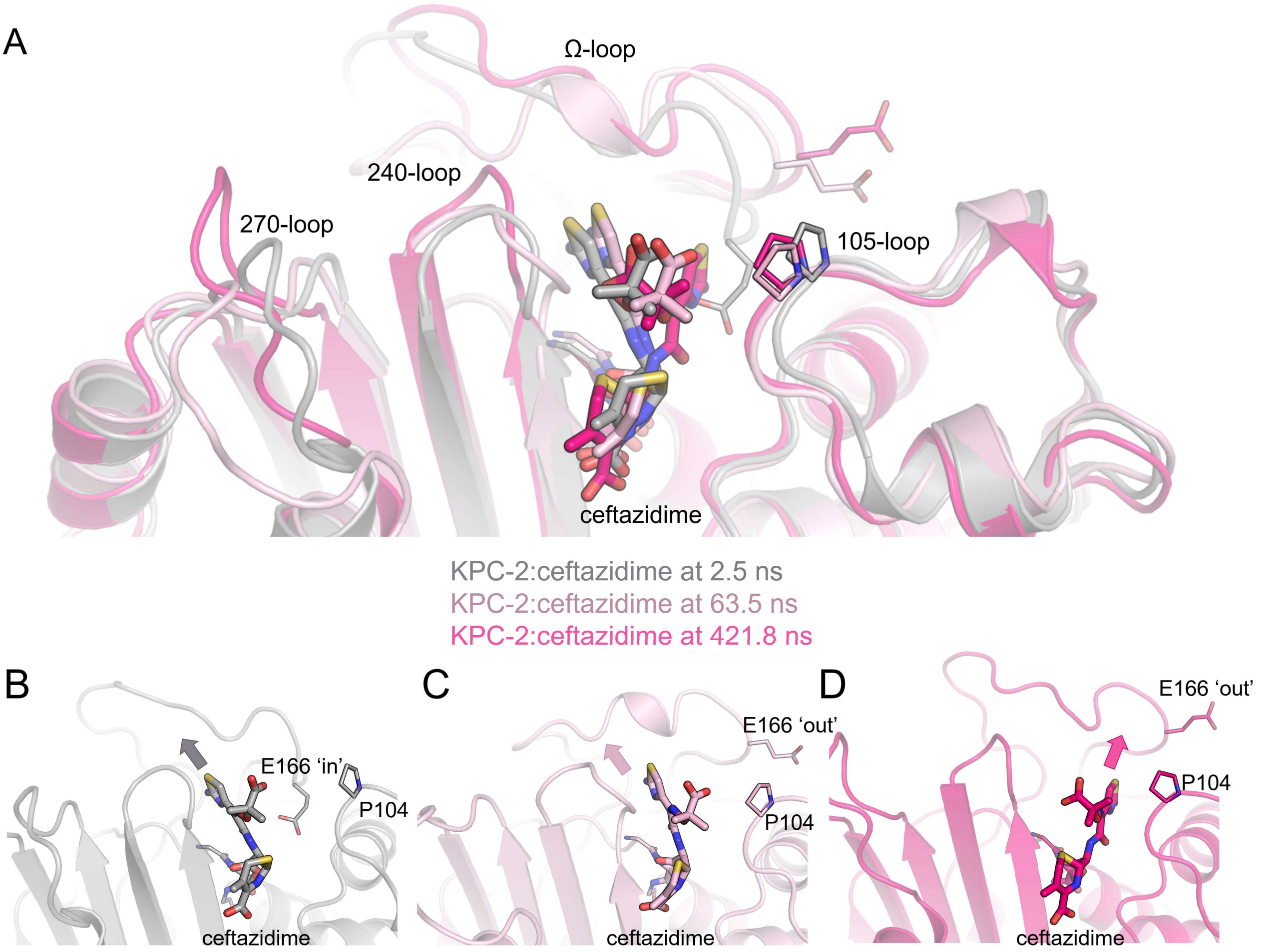

Figure S9. KPC-2:ceftazidime acylenzyme conformations during molecular dynamics simulations identified by cluster analysis. (A) Superimposition of representative frames from 2.5 ns (gray), 63.5 ns (pale pink) and 218 ns (pink). The 'flexible' 105-, 240-, 270- and Ω-loop regions identified by RMSF calculations are labelled. Close up views from the active sites are shown below with E166 and P104 shown as thin sticks, and arrows highlighting the position of the C7' aminothiazole ring (**see Figure S1D**). **(B)** 2.5 ns snapshot; **(C)** 63.5 ns snapshot (pale pink); **(D)** 421.8 ns snapshot (pink).

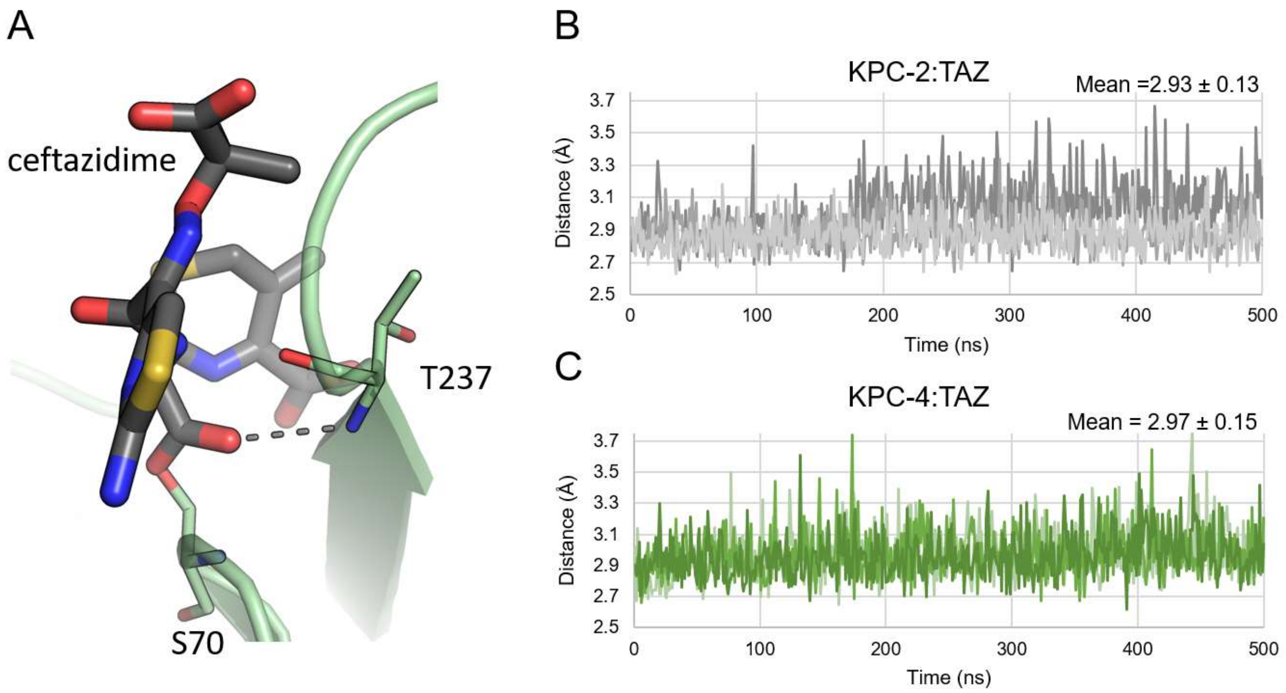

Figure S10. Positioning of the acyl-enzyme carbonyl in the oxyanion hole. The distance between the β-lactam derived carbonyl oxygen (C8=O) of ceftazidime reacted with KPC-2 and KPC-4 to the backbone nitrogen of T237 was calculated over MD runs. **(A)** Representative view from the active site of KPC-2 showing the interaction (gray dashes) between the C8 carbonyl oxygen and backbone amide nitrogen of T237(**see Figure S1D**). **(B)** Distance over triplicate 500 ns MD of KPC-2:ceftazidime (gray). **(C)** Distance over triplicate 500 ns MD of KPC-4:ceftazidime (green). In all runs for both enzymes the carbonyl group remained positioned within the oxyanion hole.

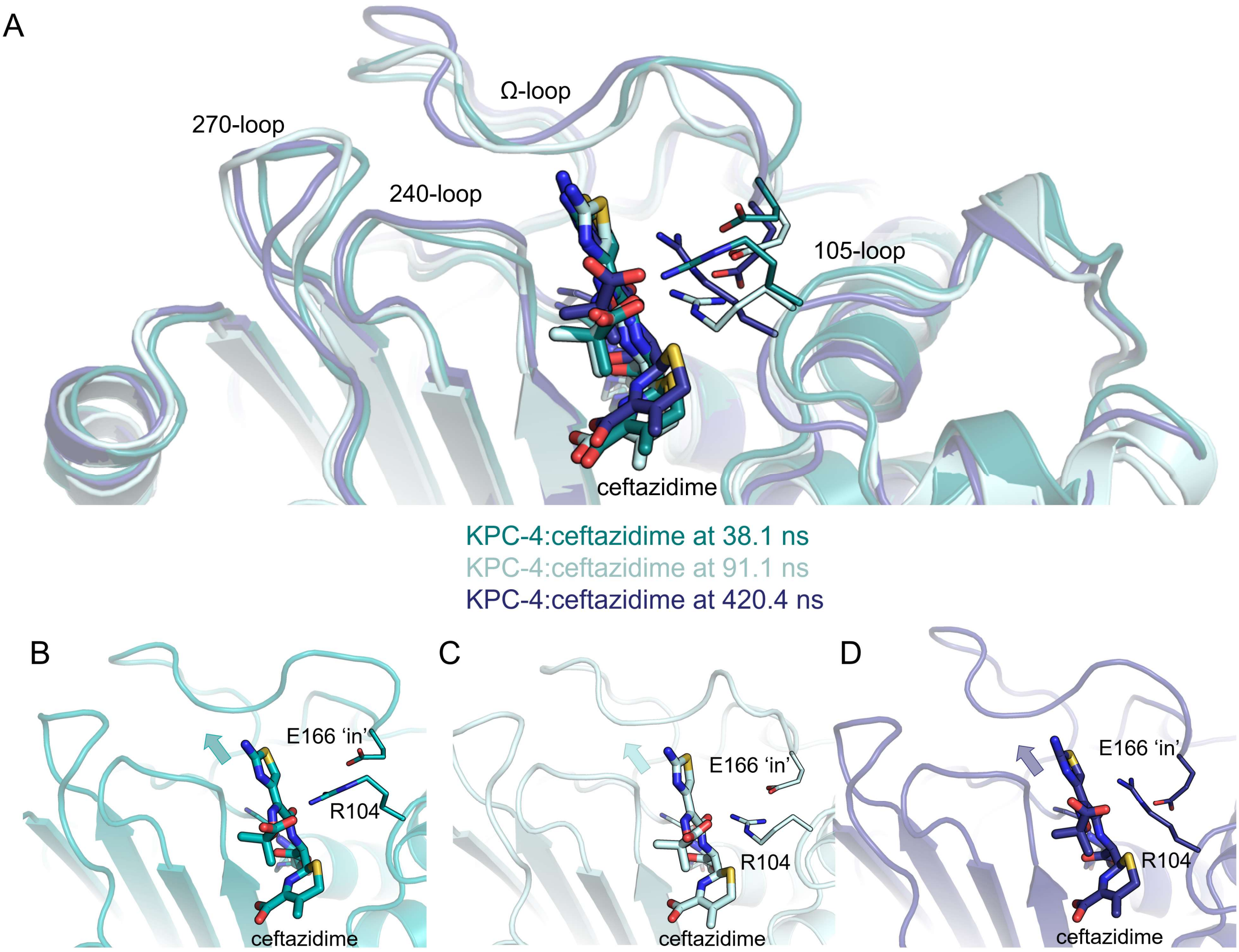

Figure S11. KPC-4:ceftazidime acyl-enzyme conformations during molecular dynamics simulations identified by cluster analysis. (A) Superimposition of representative frames from 38.1 ns (teal), 91 ns (pale blue) and 420.4 ns (dark blue). The 'flexible' 105-, 240-, 270- and Ω-loop regions identified by RMSF calculations are labelled. Close up views from the active sites are shown below with E166 and R104 shown as thin sticks, and arrows highlighting the position of the C7' aminothiazole ring (**see Figure S1D**). **(B)** 38.1 ns snapshot (teal); **(C)** 91.1 ns snapshot (pale blue); **(D)** 420.4 ns snapshot (dark blue).

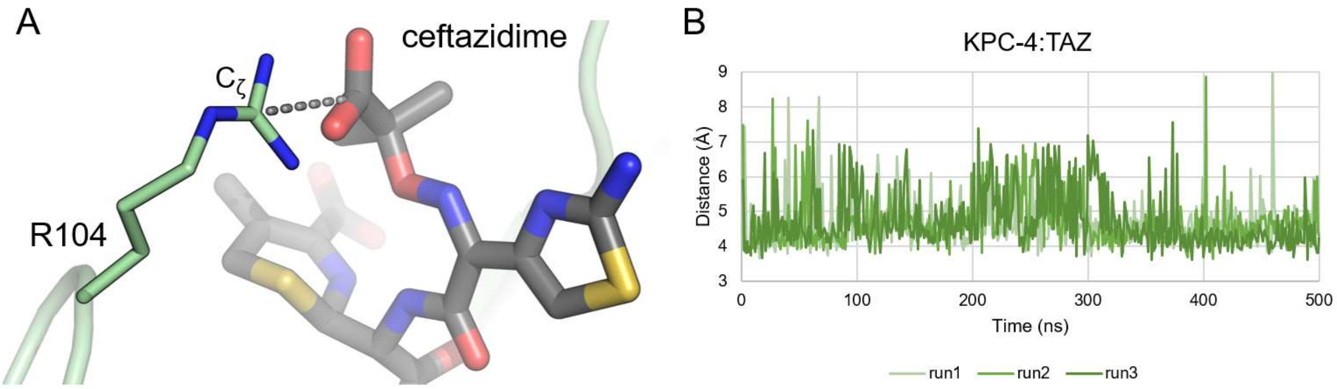

Figure S12. Interaction between R104 and the ceftazidime C7' carboxylate in the KPC-4:ceftazidime acylenzyme. Hydrogen bonds can form between either of the terminal guanidine group NH_n groups of R104 and either O of the ceftazidime carboxylate during the simulation. Here, we represent this as the distance between the C_ζ of the guanidno group of R104 and the ceftazidime carboxylate carbon. **(A)** Representative view of the measured distance during the MD simulations. **(B)** Distance plotted over the triplicate 500 ns simulations.

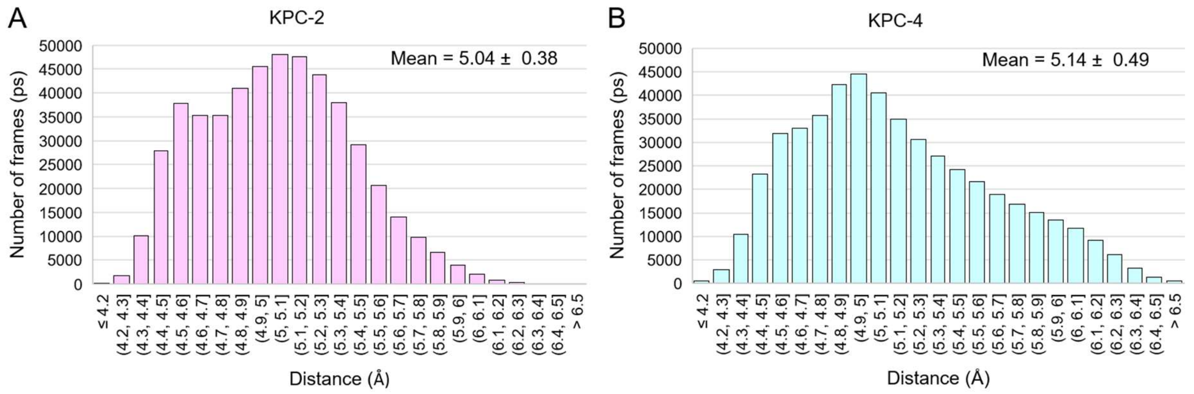

Figure S13. Distribution of distances between E166 and S70 in unliganded KPC-2 and KPC-4. Movement of the Ω-loops was analysed by measuring the distance between E166 and S70 in triplicate 500 ns trajectories of **(A)** KPC-2 and **(B)** KPC-4. The distance was averaged and plotted as histograms to represent the distribution. Each frame represents one picosecond of simulation time.

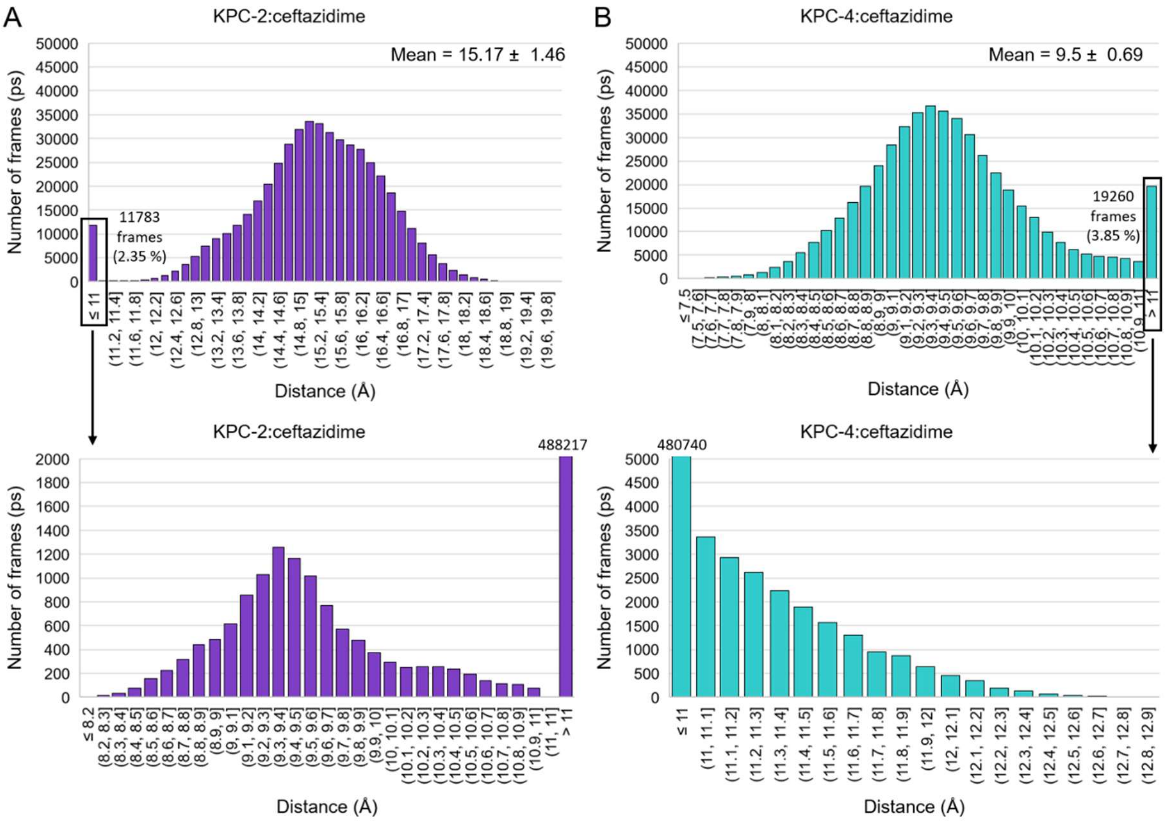

Figure S14. Ω-Loop conformations during MD simulations of KPC-2 and KPC-4 ceftazidime acyl-enzymes. Ω-Loop movement is analysed as the distribution of distances between E166 and S70 (averaged and plotted as histograms) during triplicate 500 ns trajectories of **(A)** KPC-2:ceftazidime (purple) and **(B)** KPC-4:ceftazidime (teal). Each frame represents one picosecond of simulation time. *Top*, distribution over the majority of frames (≥96% frames) in both cases. *Bottom*, distributions in the minority of frames (2.35 and 3.85 % of KPC-2 and KPC-4, respectively).

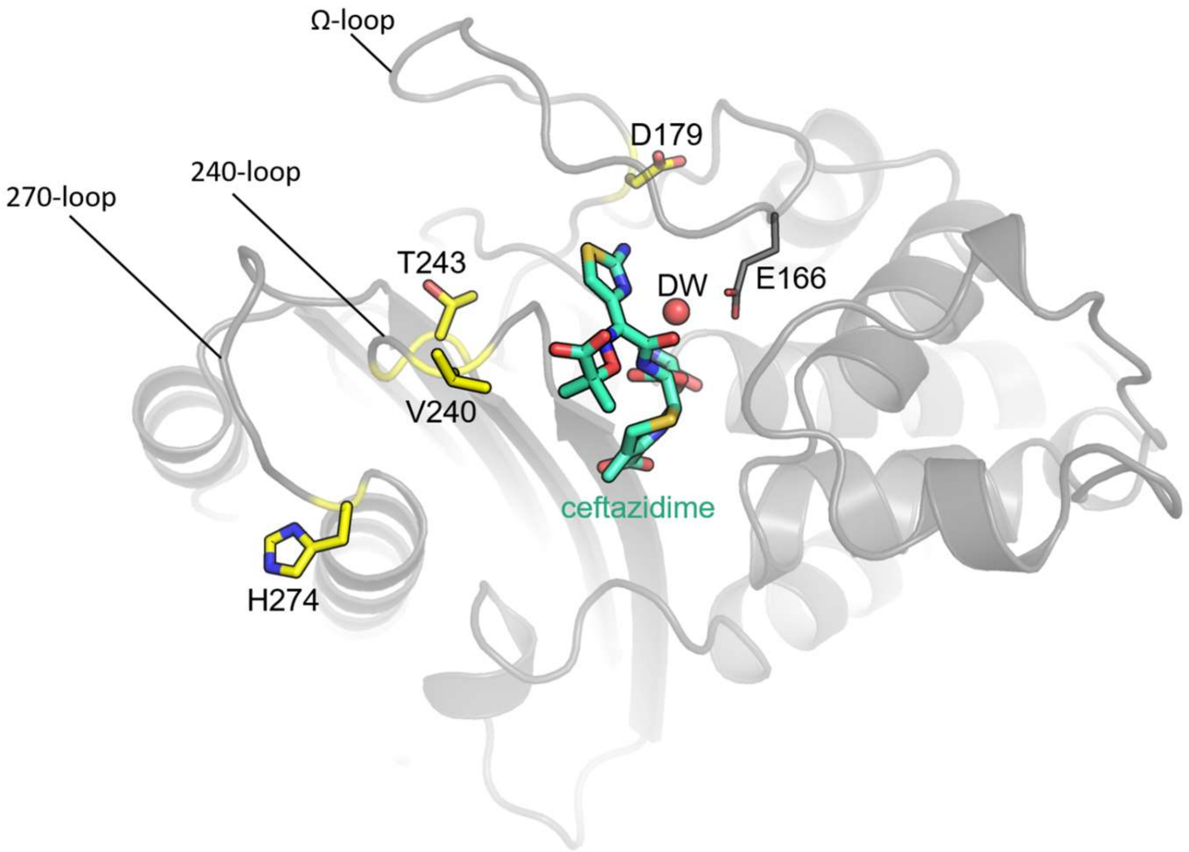

Figure S15. KPC variant positions associated with ceftazidime:avibactam resistance. Natural mutation/deletion hotspots associated with ceftazidime:avibactam resistance are labelled and highlighted in yellow within the KPC-2:ceftazidime acyl-enzyme crystal structure (with the unresolved Ω/270-loops modelled in, as described in methods).

Table S1. Uniprot and GenBank identification numbers for KPC variants.

KPC variant (alternative name)	Substitution(s)	Uniprot ID	GenBank ID*
**KPC-2 (KPC-1)**	-	Q848S6	LDDY01000008
**KPC-3**	H274Y	Q93DC4	KJ748372
**KPC-4**	P104R: V240G	I6SJK2	JMUK01000029
**KPC-5**	P104R	B0ZSP4	LPOR01000022
**KPC-6**	V240G	B2CYF2	EU555534
**KPC-7**	M49I:H274Y	-	EU729727
**KPC-8**	V240G:H274Y	B6V684	FJ234412
**KPC-9 (KPC-23)**	V240A:H274Y	-	MH450213
**KPC-10**	P104R:H274Y	C6JUP9	GQ140348
**KPC-11**	P104L	-	HM066995

* GenBank IDs obtained for each variant sourced from the β-lactamase database (1)

Table S2. Ligand occupancies, RSCC and B-factor

Structure	Occupancies (A/B)	Phenix RSCC	B-factor, Å^2 (ligand)	B-factor, Å^2 (protein)	B-factor ratio (ligand/protein)
**KPC-2^{E166Q}:cefotaxime**	0.49/0.51	0.95/0.96	32	24	1.33
**KPC-2^{E166Q}:ceftazidime**	1	0.98	17	16	1.06
**KPC-4^{E166Q}:ceftazidime**	0.75/0.25	0.98/0.98	23	18	1.28

[#]Conformers A and B if the ligand was refined as dual occupancy. B-factor is average over both conformers.

Table S3. C_α RMSDs (Å) for KPC crystal structures.

	Structure:	# of residues	1	2	3	4	5	6	7
**1**	**KPC-2 (PDB: 5ul8(2))**	271		0.157	0.097	0.201	0.165	0.592	0.278
**2**	**KPC-2^{E166Q}**	270	0.157		0.147	0.106	0.145	0.618	0.254
**3**	**KPC-4 (PDB: 6qwe(3))**	268	0.097	0.147		0.185	0.147	0.594	0.237
**4**	**KPC-4^{E166Q}**	268	0.201	0.106	0.185		0.194	0.635	0.269
**5**	**KPC-2^{E166Q}:cefotaxime**	268	0.165	0.145	0.147	0.194		0.587	0.220
**6**	**KPC-2^{E166Q}:ceftazidime**	258	0.592	0.618	0.594	0.635	0.587		0.601
**7**	**KPC-4^{E166Q}:ceftazidime**	270	0.278	0.254	0.237	0.269	0.220	0.601	

Table S4. Distances (Å) of potential H-bond interactions within unliganded KPC-2^{E166Q} and KPC-4^{E166Q}.

	KPC-2	KPC-2^{E166Q}	KPC-4	KPC-4^{E166Q}
'E/Q166:OE1' – 'N170:OD1/ND2'	2.8	2.7	2.8	4.0
'E/Q166:N/O' – 'DW'	2.5	2.3	2.5	-
'E/Q166:N/O' – 'N132:O'	3.1	3.1	3.1	3.1
'N170:O' – 'DW'	2.6	2.7	2.6	2.7
'S70:N' – 'DW'	3.2	3.0	3.1	2.9
'S70:O' – 'DW'	2.9	2.7	2.8	3.0/2.7
'N132:O' – 'K73'	2.7	2.6	2.6	2.6

References

1. Naas, T., Oueslati, S., Bonnin, R. A., Dabos, M. L., Zavala, A., Dortet, L., Retailleau, P., and Iorga, B. I. (2017) Beta-lactamase database (BLDB) – structure and function. *Journal of Enzyme Inhibition and Medicinal Chemistry* **32**, 917-919
2. Pemberton, O. A., Zhang, X., and Chen, Y. (2017) Molecular Basis of Substrate Recognition and Product Release by the Klebsiella pneumoniae Carbapenemase (KPC-2). *J Med Chem* **60**, 3525-3530
3. Tooke, C. L., Hinchliffe, P., Lang, P. A., Mulholland, A. J., Brem, J., Schofield, C. J., and Spencer, J. (2019) Molecular Basis of Class A β-lactamase Inhibition by Relebactam. *Antimicrob Agents Chemother*, AAC.00564-00519
